# Modeling the Spatiotemporal Spread and Control of African Swine Fever in the Republic of Korea Using a Patch‐Based Stochastic Framework

**DOI:** 10.1155/tbed/9997936

**Published:** 2026-05-25

**Authors:** Changdae Son, Yongin Choi, Hyojung Lee

**Affiliations:** ^1^ Department of Statistics, Kyungpook National University, Daegu, 41566, Republic of Korea, knu.ac.kr; ^2^ Research Institute of Applied Statistics, Sungkyunkwan University, Seoul, 03063, Republic of Korea, skku.edu

**Keywords:** African swine fever, patch-based stochastic modeling, seasonal transmission dynamics, spatial risk assessment, spatiotemporal dynamics

## Abstract

African swine fever (ASF) in the Republic of Korea is sustained mainly by transmission among wild boars. Wild boars account for approximately 98% of detected carcasses. Outbreaks occur mostly during the cold season (November–February), which coincides with the wild boar breeding period. Despite extensive control measures, including fence installation and hunting, ASF has continued to spread southward. This study aimed to estimate spatial outbreak risk and evaluate the effects of intervention by identifying high‐risk areas. We developed a patch‐based stochastic model that combines seasonal ASF transmission dynamics with wild boar movement. We express the wild boar movement in the model based on habitat suitability estimated from a species distribution model (SDM) that used 15 environmental variables. Using this model, we estimated outbreak probabilities for the 2023–2024 periods and classified them into four spatial risk levels: high, mid, low, and negligible. High‐risk areas accounted for 62.14% of reported carcass detections in 2023 and 81.43% in 2024. When mid‐ and low‐risk areas were included, the overall coverage exceeded 90%. The spatial distribution of high‐risk areas changed between years. High‐risk areas were concentrated in Chungbuk in 2023 and in Gyeongbuk in 2024. This pattern is consistent with the observed southward spread of ASF. The model also reproduced seasonal transmission dynamics. The reproduction number was approximately 1.33 during the increase phase from November to January and decreased to about 0.89 during the following decline phase. In addition, intervention analyses showed that movement restriction reduced the number of high‐risk patches by up to 61.7% in 2023 and 46.4% in 2024 under high‐intensity restrictions. Overall, the proposed method provides probabilistic risk assessment at a fine spatial resolution and supports quantitative evaluation of spatially targeted ASF control strategies.

## 1. Introduction

African swine fever (ASF) is a highly contagious viral disease affecting domestic pigs (*Sus scrofa domesticus*) and wild boars (*Sus scrofa*). First reported in Kenya in 1921, ASF remained predominantly endemic in sub‐Saharan Africa, and earlier incursions into Western Europe (including Italy and France) were largely eradicated [1]. However, since its introduction into Georgia in 2007 [2], the disease has spread extensively across Eastern Europe and Asia and, owing to its rapid transboundary spread and devastating impact on swine populations, is listed as a notifiable disease by the World Organisation for Animal Health. ASF virus (ASFV) is transmitted through direct contact and via contaminated body fluids, and its persistence in infectious carcasses enables prolonged environmental transmission even in the absence of active hosts [3, 4]. In the absence of effective vaccines or treatments, highly virulent strains typically cause peracute or acute disease with mortality approaching 100% within days [5, 6]. By contrast, lower‐virulence variants have been reported in some countries, including China and Vietnam [7, 8], where attenuated clinical signs, subacute or chronic courses, and substantially reduced case fatality (~30%‒70%) have been observed [9].

ASF spreads through multiple interacting pathways. Human‐mediated movement, including transport of animals and animal products and contamination, can introduce infection into distant regions; wildlife movements facilitate spread across broader spatial scales; and environmental contamination enables indirect transmission [10–12]. Among these, wild boar mobility is a principal driver of spatial spread across landscapes [5, 13]. Although wild boars generally occupy stable home ranges, long‐distance dispersal becomes particularly prevalent during the breeding season [14, 15].

Seasonal increases in wild boar mobility are primarily governed by ecological factors, including the need to locate resource‐rich habitats that support reproduction, increased food availability, and a behavioral tendency to avoid human disturbance [16]. This heightened movement during the mating season and colder months leads to elevated contact rates and greater potential for disease spread to previously unaffected areas. In addition, intensified hunting activity and increased virus stability in low temperatures further contribute to the seasonal dynamics of ASF transmission [17–19].

In the Republic of Korea, ASF was first detected on domestic pig farms in the northern regions in September 2019 [20], followed by outbreaks in wild boar populations the following month [21]. In response, the government introduced multiple control measures to prevent the transmission by wildlife, including targeted reduction of wild boar populations, rapid removal of infected carcasses [22], and large‐scale installation of physical barriers to restrict wild boar movement [23]. Fence installation commenced in November 2019, with initial deployment in Gyeonggi and Gangwon provinces, followed by additional expansion within Gangwon, and was subsequently extended through Chungbuk and Gyeongbuk across six implementation phases (Phases 1 to 5–2). Overall, 1831 km of fencing was constructed across 34 administrative districts, spanning Gangwon (1179 km), Gyeonggi (352 km), Chungbuk (63 km), and Gyeongbuk (237 km), with an estimated total cost of approximately KRW 113 billion [24]. Despite these extensive efforts, the disease has continued to expand southward, resulting in 3409 reported cases as of December 2024 [25]. Specifically, most cases were concentrated in Gyeongbuk, Gangwon, and Chungbuk, which collectively accounted for 2810 of 3409 cases (82.48%), with clinical–epidemiological features consistent with outbreaks caused predominantly by highly virulent ASFV strains [26–29]. As these provinces are situated along the major ASF transmission route of Korea from north to southeast, they have been identified as focal areas of ASF transmission, warranting closer investigation to better understand the spatial dynamics and develop targeted intervention strategies.

The Korean ASF epidemic primarily affects wild boars, accounting for 98.40% of all reported cases, underscoring wildlife as the main reservoir and driver of spread. One contributing factor is the distinctive pig‐farming system in Korea: Unlike some European countries with free‐range production, domestic pigs in Korea are typically housed in enclosed, high‐biosecurity indoor facilities that limit interactions with external sources of infection [30]. Stringent containment protocols, including mandatory culling of all pigs on infected farms within 1–2 days of detection, have further minimized secondary outbreaks in the domestic pig sector [23]. Consequently, the ecological and spatial understanding of wild boar movement is central to ASF control. Regions with dense forests and abundant food resources typically harbor higher wild boar populations, increasing the local transmission risk [31]. In contrast, fragmented habitats and artificial barriers, such as roads or fences, can impede movement and influence spatial outbreak patterns [32].

Furthermore, ASF activity in Korean wild boar exhibits pronounced seasonality, with detections increasing in late autumn, typically peaking around November, and remaining elevated through early spring (approximately November−April) [31]. During 2020–2022, monthly incidence was ~3.8 times higher in the cold season, which accounted for nearly 80% of all reported cases [33], consistent with winter–spring transmission clusters and a scoping review of 2019–2021 data [34, 35]. Longer carcass persistence in cold conditions—which extends both detectability and environmental maintenance of infection [36]—offers a plausible mechanistic explanation and underscores the need for spatially explicit models that integrate outbreak data with wild boar ecology and seasonal transmission patterns for risk assessment.

Existing studies on ASF have yielded valuable insights into critical aspects of the disease, including the role of infected carcasses in sustaining transmission [37, 38], influence of habitat structure and movement pathways on regional spread [39], impact of population density‐dependent contact rates on transmission dynamics [40], as well as the intervention strategies assessed through scenario analysis [41, 42]. Spatially explicit ASF transmission models, such as those by Taylor et al. [43] and Dankwa et al. [44], have been developed to explore spread across heterogeneous environments and evaluate control strategies. However, many of these models have been formulated under simplified or data‐limited assumptions. Meanwhile, studies analyzing ASF outbreaks in Korea have largely employed descriptive analyses [34, 45–47], which characterize observed patterns rather than explicitly model infection processes or quantify stochastic spatial propagation.

These gaps highlight the need for an ecologically informed transmission model that accounts for wild boar activity patterns to explain the spatiotemporal dynamics of ASF. This study aimed to develop a patch‐based stochastic modeling framework that captures ASF spread arising from wild boar movement using Korean surveillance data from 2019 to 2024. The framework quantifies patch‐level outbreak probabilities to characterize outbreak propagation during the 2023–2024 outbreaks and delineate high‐risk areas across the landscape. Within this framework, we further evaluate the impacts of movement‐restriction interventions designed to emulate fence‐like containment across ecologically connected regions.

## 2. Materials and Methods

**Figure 1:**
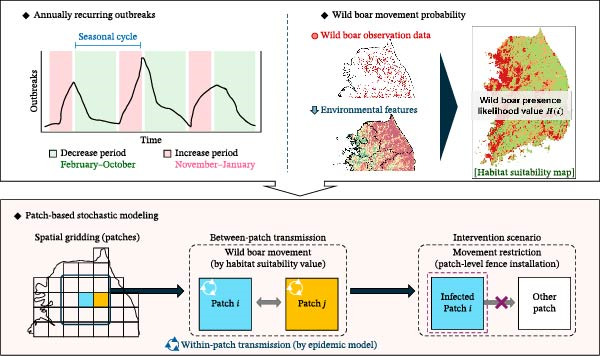
Overview of patch‐based stochastic modeling.

We developed a probabilistic framework to simulate the spatiotemporal spread of ASF in Korea by integrating epidemiological surveillance with ecological determinants. The model combines seasonally varying transmission dynamics and habitat‐driven dispersal probabilities derived from species distribution modeling, enabling patch‐based stochastic simulation of both within‐ and between‐patch transmission. Subsequently, scenario analyses were conducted to evaluate the potential effectiveness of movement‐restriction and population‐reduction strategies (Figure 1).

### 2.1. Data Description

To construct the modeling framework, we assembled ASF‐related datasets covering outbreak records, wild boar ecology, environmental covariates, and intervention measures (Table 1). These datasets were harmonized at a weekly temporal scale and integrated into the spatial simulation model. Further details are available in Appendix B.1.

**Table 1:** Summary of the data categories and data sources.

Category	Data description
Epidemiological data	ASF outbreak data, including ASF‐positive carcass records, were obtained from the Wildlife Disease Information System (WADIS) for 2019–2024 [25]. Annual wild boar hunting rates and habitat density estimates were retrieved from the Ministry of Environment [48, 49].
Wild boar distribution data	Wild boar occurrence records collected through the National Institute of Ecology (NIE) monitoring programs (2019–2023) [49] were used as the basis for constructing the habitat suitability model.
Topographic data	Altitude (i.e., elevation) and slope (including slope angle and slope aspect angle) data were sourced from the National Geographic Information Institute (NGII) at a 5 m × 5 m spatial resolution [50], characterizing landscape heterogeneity relevant to wild boar habitats.
Climatic data	Annual mean, minimum, and maximum temperature, along with precipitation data, were obtained from the Korea Meteorological Administration (KMA) [51]. It accounts for seasonal and environmental effects influencing ASF persistence and wild boar activity.
Ecological data	Distances to natural features, including forests and rivers, were derived from the European Space Agency (ESA, 2021) land cover dataset (10 m × 10 m resolution) [52]. These features were included to quantify habitat accessibility and connectivity.
Human‐related data	Human population density was obtained from WorldPop (2020) at 1 km × 1 km resolution [33]. Distances to cropland and built‐up areas were extracted from the ESA dataset to represent human‐related effects [52].

### 2.2. Epidemic Model for the ASF‐Infected Carcasses Trend

To characterize nationwide ASF transmission dynamics and quantify seasonal variation, we developed an epidemic model by using carcass surveillance data from 2019 to 2024. We aim to estimate time‐varying transmission rates that reflect seasonal fluctuations.

#### 2.2.1. Epidemiological Data

Daily ASF‐positive carcass reports were retrieved from the National Institute of Wildlife Disease Control and Prevention (Wildlife Disease Information System) for the period 2019–2024 [25]. The dataset comprised three observation types (carcasses, hunted animals, and trapped animals) with 4169 ASF‐positive records in total: 3710 carcasses (88.99%), 399 hunted (9.57%), and 60 trapped (1.44%). To maintain consistency in the observation process and reduce potential biases from control activities, analyses focused on carcass records.

According to the outbreak situation reports [53, 54], a subset of ASF detections reported during 2023–2024 in Busan and northern Gyeonggi, Korea, were attributed to human‐mediated transmission. Additionally, detections reported in nearby areas during the same periods were interpreted as likely secondary spread associated with these events. Consequently, 31 records (2 in 2023 and 29 in 2024) were excluded to ensure that the analysis reflected natural wild boar–driven transmission. Therefore, we aggregated daily ASF‐positive wild boar carcass reports into weekly counts.

**Figure 2:**
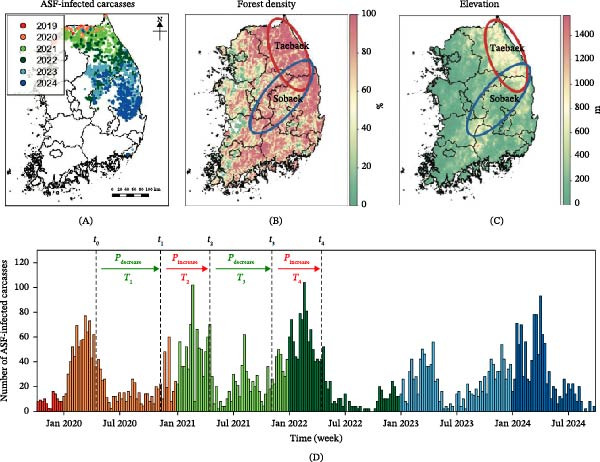
ASF epidemiological data in Korea (2019–2024). (A) Geographic distribution of ASF‐infected wild boar carcass detection sites. (B) Forest density and (C) elevation across Korea, with the Taebaek and Sobaek mountain ranges highlighted in red and blue, respectively. (D) Monthly counts of ASF‐infected carcass reports, with seasonal epidemic phases categorized into increase and decrease phases.

Figure 2 summarizes the key empirical patterns. The spatial distribution of ASF‐positive carcasses (Figure 2A) followed major mountain ranges such as the Taebaek and Sobaek, which extend from northeastern Korea toward the central and southern inland regions (Figure 2B,C) [31, 55]. This spatial alignment reflects the ecological context of Korea, where most reported cases originate from wild boar–driven infections and closely correspond to boar habitats, such as forest‐dense and high‐elevation regions. Temporally, ASF carcass counts exhibited a clear seasonal pattern, with higher detections during the colder months, broadly aligning with ecological patterns such as increased wild boar mobility during the breeding seasons (approximately November–April) and prolonged carcass persistence at low temperatures (Figure 2D) [18, 19, 22, 34, 56–58]. These empirical features informed the seasonal phase structure, defined as an increase phase (*P*_increase_), marked by a rise in ASF cases, and a decrease phase (*P*_decrease_), during which case numbers typically declined.

#### 2.2.2. Epidemic Model

We employed a deterministic susceptible–infectious–carcasses–reported (SICR) compartmental model to describe ASF transmission dynamics in wild boar populations. A deterministic SICR model was adopted to facilitate parameter estimation from carcass surveillance data. The model consists of four epidemiological states: susceptible (*S*), infectious (*I*), carcasses of infected wild boars that died but remain infectious (*C*), and reported carcasses (*R*). A recovered state was excluded because highly virulent ASFV strains circulating in Korea are almost always fatal. Population dynamics evolve at weekly time steps *t* as follows:

St+1=St+b Popt−βtStIt+θCtPopt−μ+μpSt,

It+1=It+βtStIt+θCtPopt−μ+μpIt−γIt,

Ct+1=Ct+μ+γIt−fδtCt−1−fδctCt,

Rt+1=Rt+fδtCt,

where Pop(*t*) = *S*(*t*) + *I*(*t*) denotes the active wild boar population available for contact and transmission at time *t*. Hunting‐induced deaths, *μ*_*p*_(*S*(*t*) + *I*(*t*)), are assumed to remove wild boars immediately and therefore do not generate carcasses; thus, hunting‐induced deaths do not contribute to the ASF‐infected carcasses (*C*).

Time‐varying parameters include the transmission rate *β*(*t*), capturing seasonal changes in overall transmission intensity, and *δ*_*c*_(*t*) refers to the carcass infectiousness‐loss rate. *δ*_*c*_(*t*) is defined as *δ*_*c*_(*t*) = *δ*_0,*c*_exp(−*s* cos^2^(*π*(*t* − *ϕ*)/*T*)), where *T* = 52 weeks represents the annual cycle, *δ*_0,*c*_ is the baseline loss rate, *s* is the seasonal amplitude, and *ϕ* indicates the phase that aligns the seasonal pattern [40]. Detailed descriptions and parameter values for *δ*_*c*_(*t*) are provided in Appendix A.1 and Table S1. Under this definition, *δ*_*c*_(*t*) yields lower values during colder periods (corresponding to longer carcass infectivity periods, 1/*δ*_*c*_(*t*)) and higher values during warmer periods, thereby capturing seasonal variation in carcass‐mediated transmission [18, 58].

To quantify transmission potential over time, we computed the effective reproduction number *R*_*t*_ from the system using the next‐generation matrix method [59, 60]: $R_{t}=\frac{S\left(t\right)}{\text{Pop}\left(t\right)}\left(\frac{\beta \left(t\right)}{\gamma +\mu +\mu_{p}}+\frac{\beta \left(t\right)\theta }{f\delta \left(t\right)+\left(1-f\right)\delta_{c}\left(t\right)}\right)$. This quantity reflects the number of secondary infections generated by an infectious wild boar in a population comprising both susceptible and nonsusceptible hosts. Further details on *R*_*t*_, along with model variable descriptions, are available in Appendix A.2

#### 2.2.3. Estimation of Seasonal Transmission Dynamics

To estimate the time‐varying transmission rates *β*(*t*), we calibrated the SICR model to weekly carcass counts observed between October 2019 and September 2022 (156 weeks). We adopted a least‐squares optimization approach, minimizing the discrepancy between observed counts of ASF‐infected wild boar carcasses (*C*_observed_(*t*)) and model‐predicted (*C*_predict_(*t*)) carcass counts, defined as *C*_predict_(*t*) = *f* *δ*(*t*)*C*(*t*), where *f* denotes the detection rate for carcasses and *δ*(*t*) represents the reporting rate accounting for the delay time between the death of a wild boar and the detection of its carcass through surveillance.

The first seasonal cycle starts at a turning point *t*_0_ ∈ [1, 51], marking the onset of the decrease phase, while the preceding interval [0, *t*_0_ − 1] was treated as the increase phase. To formalize this seasonal structure over the 3‐year simulation period, we defined the transmission rate *β*(*t*) as a piecewise constant periodic function, alternating between decreasing and increasing phases within each annual cycle. Specifically, each year comprises an increase phase of fixed duration *ω* weeks (*T*_1_ = [0,  *t*_0_ − 1], *T*_*n*_ = [*t*_0_ + 52(*n* − 1) − *ω*,  *t*_0_ + 52(*n* − 1) − 1] for *n* ≥ 2), followed by a decrease phase lasting 52 − *ω* weeks (*ω* = 1,  2,   … ,  51). Accordingly, the sets of increase and decrease phases across the three annual cycles were defined as follows:

Pincrease=t∈∪i=13Ti,Pdecrease=t∉Pincrease.

The parameter *β*(*t*) captures transmission intensities during these respective phases:

βt= βincrease  if t∈Pincreaseβdecrease if t∈Pdecrease.

The objective function (*L*) for parameter estimation was formulated as follows:

Lβincrease,  βdecrease, ω, t0=∑t=0TCpredictt; βincrease,  βdecrease, ω, t0−Cobservedt2.

The estimated parameter set $\left(\beta_{\text{increase}}^{*},\beta_{\text{decrease}}^{*},\;\omega^{*},\;t_{0}^{*}\right)$ was thus obtained by solving the following optimization problem.

βincrease∗,βdecrease∗, ω∗, t0∗=argminβincrease,βdecrease,ω,t0Lβincrease, βdecrease, ω, t0.

The estimation relied on epidemiological and surveillance inputs, which were defined as follows. The total wild boar population was scaled to 229,259 individuals, calculated by multiplying a density of 2.3 boars per km^2^ multiplied by a national land area of 99,678 km^2^, using national monitoring data from the NIE [49]. This estimate was then used to define the initial population size, Pop(0). The initial epidemiological variables were subsequently initialized at simulation time *t* = 0 (October 7, 2019) on the basis of ASF‐positive carcass records. Let Obs(*t*) denote the number of ASF‐positive carcasses reported at simulation time *t*. After accounting for a reporting delay time (~5 weeks) and a carcass detection rate (*f* = 0.25), the initial conditions were specified as $C\left(0\right)=4\times \sum_{t=1}^{5}\text{Obs}\left(t\right)$, *I*(0) = *C*(0), *R*(0) = 0, and *S*(0) = Pop(0) − *I*(0). Further details of the population scaling and initialization procedures are provided in Appendix A.3.

### 2.3. Spatiotemporal ASF Risk Assessment

We developed a patch‐based stochastic modeling framework that integrates local epidemic dynamics with between‐patch movement of infectious wild boars to reconstruct observed spatial expansion patterns and quantify regional variations in transmission risk. Using this structure, we simulated ASF spread over two outbreak periods, November 2022–September 2023 (2023 outbreak) and November 2023–September 2024 (2024 outbreak), respectively.

#### 2.3.1. Habitat‐Based Dispersal Probability for Movement Between Patches

To model spatial transmission driven by wild boar movement, we discretized the Korean territory into uniform spatial units, each corresponding to a 10 km × 10 km grid cell (patches). Within this framework, 76, 191, and 208 patches were located in Chungbuk, Gangwon, and Gyeongbuk, respectively, where most ASF cases occurred. For patch‐level model simulation, weekly ASF‐positive data (defined in Section 2.2.1) were allocated to patches according to detection locations to construct patch‐level weekly data.

The spatial resolution was selected to align with typical wild boar home range sizes (2–10 km^2^ [61, 62]). This resolution permits within‐patch movement to capture local transmission processes while constraining between‐patch movement to ecologically realistic distances [63, 64]. Long‐distance dispersal was represented as movement between patches and, within a single time step, was limited to adjacent patches under an eight‐neighbor structure. This constraint avoids implausibly long jumps across distant regions. Moreover, the selected spatial scale is consistent with the Korean ASF standard operating procedures (SOPs), which specify a 10‐km control radius around detected outbreak sites [23].

Given that wild boars tend to move preferentially toward areas offering more favorable environmental conditions [14, 15, 65, 66], we estimated habitat suitability for each patch using a Maximum Entropy (Maxent) algorithm‐based species distribution model (SDM). The SDM was trained on wild boar occurrence records collected between 2019 and 2023 [67], incorporating topographic, climatic, ecological, and human‐related variables [33, 50–52].

Given the availability of presence‐only data, pseudo‐absence points were generated throughout Korea and treated as absence locations. These points were randomly sampled across Korea at a 1:3 ratio relative to presence records [68, 69]. Model performance was assessed using standard discrimination metrics to determine whether the SDM provided a robust representation of wild boar habitat suitability. The area under the receiver operating characteristic curve (AUC) quantified overall discrimination ability between presence and absence across all thresholds, whereas sensitivity and specificity measured correct classification of presence and absence, respectively. The true skill statistic (TSS) further summarized classification performance by integrating errors from both missed presence locations and incorrectly included nonoccurrence areas. The resulting suitability surface was subsequently aggregated by averaging suitability values within each patch. Detailed model specifications and performance evaluations are presented in Appendix B.2.

**Figure 3:**
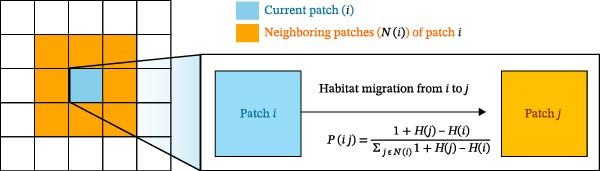
Movement framework between patches considering habitat suitability.

Patch‐level habitat suitability values were then used to define the movement probability between adjacent patches following Taylor et al. [43]. Specifically, the probability of movement from patch *i* to a neighboring patch *j* ∈ *N*(*i*), where *N*(*i*) represents the set of neighbors around patch *i*, was defined as follows:

Pi→j=1+Hj−Hi∑k∈Ni1+Hk−Hi,

where *H*(*i*) and *H*(*j*) denote habitat suitability values for the current and adjacent patches, respectively. The term (1 + *H*(*j*) − *H*(*i*)) represents the relative gain in suitability associated with moving toward patch *j*, reflecting the tendency of wild boars to preferentially move toward environmentally attractive locations. This formulation incorporates local habitat gradients into the stochastic movement process, providing a biologically meaningful representation of suitability‐driven spread across the patch network. A schematic representation of this approach is illustrated in Figure 3.

#### 2.3.2. Patch‐Based Stochastic Modeling Structure for Spatial Spread

**Figure 4:**
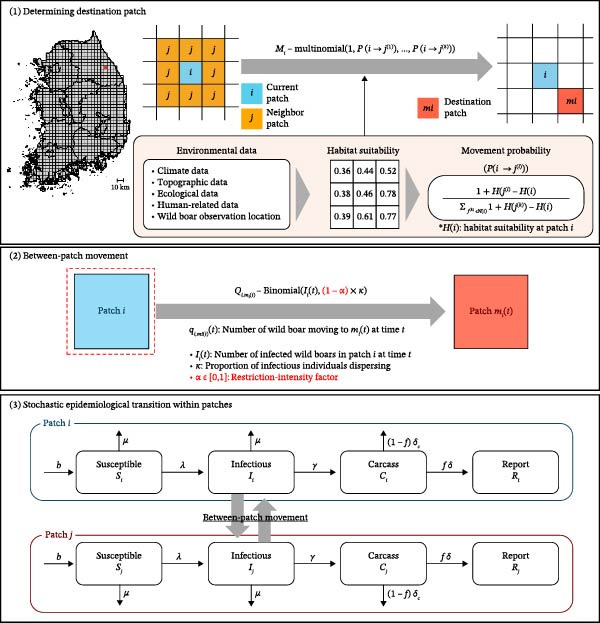
Schematic diagram of the patch‐based transmission framework. (1) Infectious individuals select destination patches based on habitat suitability. (2) Some individuals move between patches beyond their typical home ranges. (3) Stochastic epidemiological transitions are simulated within each patch. All notations are provided in Table S2.

Figure 4 presents a schematic overview of a patch‐based stochastic modeling structure that integrates local epidemic dynamics within patches with movement between patches driven by habitat suitability, thereby representing spatial ASF transmission across Korea. The model advances in time steps and, at each step, implements three sequential processes: movement–destination determination, between‐patch movement simulation, and within‐patch epidemiological state transitions.

1. Determining destination patches: For each patch *i*, infectious wild boars probabilistically select a destination among its adjacent neighbors based on the movement probabilities defined in Section 2.3.1. These probabilities reflect the ecological tendency of wild boars to move toward patches with higher habitat suitability [4]. For each time *t*, the destination *m*_*i*_(*t*) is randomly sampled from a multinomial distribution of M_i_:
Mi∼Multinomial1, Pi→j1, Pi→j2,…, Pi→j8,
  where *P*(*i* → *j* ^(*l*)^) denotes the suitability‐based probability of movement from patch *i* to its adjacent neighboring patch *j* ^(*l*)^ for *l* = 1,  2,   … ,  8.
2. Between‐patch movement: Empirical observations indicate that a subset of wild boars, particularly during the breeding season, engage in long‐range movement beyond their typical home ranges [57]. Previous studies have reported that ~10%–20% of individuals exhibit this dispersal behavior, with a point estimate of 15.4% [70]. To incorporate this behavior, we introduced a dispersal adjustment factor (*κ* = 0.154), representing the estimated proportion of infectious individuals capable of moving beyond their typical ranges. For each patch *i* at time *t*, the number of infectious individuals engaging in long‐range movement was modeled as follows:
Qi, mit∼BinomialIit, κ,
  where *I*_*i*_(*t*) represents the number of infectious individuals in patch *i* at time *t*, and *m*_*i*_(*t*) denotes the destination patch assigned to infectious individuals moving outward from patch *i* at time *t*. This step governs the spatial distribution of infection pressure across the patch network.
3. Stochastic epidemiological transitions within patches: Within each patch, newly infected cases are assumed to be Poisson random variables. The number of new infections in patch *i* arises from local contact with infectious individuals (*I*_*i*_(*t*)), carcass‐mediated environmental transmission (*C*_*i*_(*t*)), and infectious individuals dispersed from neighboring patches:
Infectit∼PoissonβtSitIit+θCit−qi, mitt+∑j∈Niqj,itPopit,
  where *β*(*t*) is the seasonal transmission rate, *c* the relative carcass infectiousness, and Pop_*i*_(*t*) is the total population in patch *i* at time *t*. The terms *q*_*j*,*i*_(*t*) and $q_{i,m_{i}\left(t\right)}\left(t\right)$ represent incoming and outgoing infectious individuals, respectively. ASF‐induced mortality among infectious wild boars was modeled as follows:
ASF-induced deathit∼PoissonγIit,
  where *γ* is the ASF‐induced mortality rate. Carcasses are subsequently either reported through surveillance activities (detection *f* with reporting rate *δ*(*t*)) or naturally decomposed (at rate 1/*δ*_*c*_(*t*)):
Reportit∼PoissonfδtCit,and Lossit∼Poisson1−fδctCit,

Beyond disease‐induced transitions, the model includes demographic processes (birth and natural mortality) and management interventions (such as hunting):

Birthit∼Poissonb Popit,

Natural deathX, it∼Poissonμ Xit for X∈S, I,

HuntX, it∼PoissonμpXit for X∈S, I,

Here, *b* represents the per capita birth rate (four offspring per adult per year, consistent with observed ranges of 3–10 [42, 57, 71, 72]), and *μ*_*p*_ is the hunting removal rate, parameterized based on Korea’s ASF control target of a 70% annual population reduction, based on national hunting records from 2019 to 2024 [49].

#### 2.3.3. Estimation of Risk Areas Using the Patch‐Based Stochastic Model With Movement

Building on the stochastic modeling framework described above, we evaluated the spatiotemporal spread of ASF across Korea by linking seasonally varying local transmission with the movement of infected wild boars based on habitat suitability. This framework produces patch‐level outbreak probabilities, which are used to classify spatial risk levels and to assess how well‐predicted risk areas correspond to observed outbreak locations.

Wild boar population sizes for individual patches, denoted as Pop_*i*_(0) for patch *i*, were compiled by distributing the scaled national population across patches in proportion to habitat suitability. Given the resulting patch‐level population sizes, epidemiological states were initialized for each outbreak period (2023 and 2024) using ASF‐positive carcass records within each patch. Let Obs_*i*_(*t*) denote the number of carcasses reported in patch *i* at simulation time *t*, and the initial conditions for patch *i* were specified as $C_{i}\left(0\right)=4\times \sum_{t=1}^{5}{\text{Obs}}_{i}\left(t\right)$, *I*_*i*_(0) = *C*_*i*_(0), *R*_*i*_(0) = 0, and *S*_*i*_(0) = Pop_*i*_(0) − *I*_*i*_(0). Further details of the initialization procedures are provided in Appendix A.3.

Using these initializations, we performed 100,000 stochastic realizations of spatial spread for each outbreak period. Across iterations, each patch *i* was monitored to determine whether at least one reported carcass occurred during the simulation period. Following the national ASF control SOP [23], a patch was classified as an infected patch in iteration *k* if it satisfied:

Rikt≥1 for any t,

where ${R_{i}}^{\left(k\right)}\left(t\right)$ denotes the number of reported carcasses in patch *i* at time *t* for iteration *k* ∈ {1,  2,   … ,  *K*}, with *K* = 100,000. The outbreak probability for each patch was then calculated as follows:

pi=1K∑k=1K1Rikt≥1,

where 1(⋅) is the indicator function, taking the value 1 if the patch experienced infection during iteration *k*. Specifically, $\sum_{k=1}^{K}1\left({R_{i}}^{\left(k\right)}\left(t\right)\geq 1\right)$ refers to the total number of iterations in which patch *i* was classified as infected.

• Patch‐level spatial risk classification: To delineate spatial risk areas across the landscape, each patch was assigned to one of four risk levels based on its simulated outbreak probability *p*_*i*_:
  • High risk: 0.75 ≤ *p*_*i*_  ≤ 1.
  • Mid risk: 0.25 ≤ *p*_*i*_ < 0.75.
  • Low risk: 0.01 ≤ *p*_*i*_ < 0.25.
  • Negligible: 0  ≤ *p*_*i*_ < 0.01.

This classification provides a basis for comparing model‐predicted risk patterns with observed ASF detections. To evaluate predictive performance in identifying spatial locations with elevated outbreak potential, we further examined how accurately high‐risk patches classified by the model captured observed outbreak patches. This assessment was based on region‐specific confusion matrices that distinguish correctly and incorrectly classified outbreak and nonoutbreak patches.

• Regional case coverage: To complement patch‐level spatial risk classification, we assessed how effectively predicted risk areas captured reported carcass locations within each administrative regions *g* ∈ *G* = {Chungbuk,  Gangwon,  Gyeongbuk,  Korea}. For each region (*g*) and risk level (level ∈ {low,  mid,  high}), regional case coverage (Coverage_*g*, level_) was defined as follows:
  • High‐risk coverage: the proportion of reported cases that fall within patches classified as high risk,
Coverageg, high=CasesestighighCasesobsg.
• Mid‐risk coverage: the proportion of reported cases that fall within patches classified as high or mid risk,
Coverageg, mid=Casesestigmid+CasesestighighCasesobsg.
• Low‐risk coverage: the proportion of reported cases that fall within any predicted risk area,
Coverageg, low=Casesestiglow+Casesestigmid+CasesestighighCasesobsg.

Here, ${{\text{Cases}}^{\text{esti}}}_{g}\left(\cdot \right)$ denotes the number of observed cases falling within patches classified into the corresponding risk level, and ${{\text{Cases}}^{\text{obs}}}_{g}$ denotes the total number of observed outbreak cases assigned to patches within region *g*, with repeated detections in the same patch counted separately. We further defined hot spots as region *g* containing a high concentration of predicted high‐risk patches, which are interpreted as estimated outbreak risk areas. Collectively, these regional indicators provide a quantitative basis for evaluating spatial predictive performance and regional variations in ASF transmission risk.

• Patch‐level predictive performance: We further assessed probabilistic predictive performance using the Brier score, which quantifies the accuracy of predicted outbreak probabilities without thresholding. The Brier score was defined as follows:
Brier score=1W∑i=1Wpi−1Obsi≥12,
  where *p*_*i*_ denotes the simulated outbreak probability for patch *i*, and Obs_*i*_ represents the number of detected carcasses in that patch. *W* indicates the total number of patches within a region, comprising 475 nationwide (76 in Chungbuk, 191 in Gangwon 191, and 208 in Gyeongbuk). A Brier score of 0.25 serves as a reference baseline, with lower values indicating closer correspondence between predicted outbreak probabilities and observed detections [73].
• Sensitivity analysis: We conducted sensitivity analyses to examine how the evaluated outbreak probabilities and spatial risk classification respond to key modeling assumption and parameter value variations. Two components were considered: (1) structural assumptions, including spatial resolution (from a baseline 10 km × 10 km to a finer 5 km × 5 km grid) and movement structure (ranging from adjacent‐only movement to movement across two patches); and (2) parameter values, including the initial population size for wild boars Pop(0), dispersal proportion of infectious individuals *κ*, the detection rate *f*, and the reporting delay 1/*δ*(*t*). Each parameter was increased and decreased by 50% relative to its baseline value.

#### 2.3.4. Quantifying Intervention Impacts on ASF Spread

ASF control strategies in Korea are implemented under the SOP established by the Ministry of Agriculture, Food, and Rural Affairs, which includes movement restrictions, enhanced surveillance, and wild boar population management [23]. Among these interventions, the installation of physical barriers, such as extensive fences, is one of the widely implemented measures to limit wild boar movement and mitigate long‐range transmission risk.

In this study, we implemented a patch‐level intervention framework that simulates reactive containment responses to local outbreak detection, conceptually reflecting the role of fencing. This framework allowed us to evaluate the effectiveness of spatially targeted ASF control policies aimed at suppressing disease spread through wild boar–mediated transmission pathways.

Scenario simulations were conducted by modifying the baseline wild boar movement process described in Section 2.3.2. In each scenario, movement restrictions were triggered upon detection of ASF‐infected carcasses (activated at time *t* when ${R_{i}}^{\left(k\right)}\left(t\right)\geq 1$ for patch *i* in simulation iteration *k*), in accordance with the surveillance‐based response protocol of Korea. Once triggered, movement restrictions were applied to suppress movements both into and out of an infected patch, thereby reducing dispersal between the infected patch and its neighboring patches through a restriction‐intensity factor (*α*), ranging from 0 (no restriction) to 1 (complete suppression). Under restriction, the number of infectious individuals dispersing from patch *i* to its chosen destination *m*_*i*_(*t*) was modeled as follows:

Qi,mit∗∼BinomialIit, κ1−α,

where ${q^{*}}_{i,m_{i}\left(t\right)}$ denotes the sampled value from $Q_{i,m_{i}\left(t\right)}^{*}$, which is the number of infectious boars moving under restriction ((1 − *α*)), and *κ* is the baseline dispersal adjustment factor described in Section 2.3.2.

In all scenarios, patches were classified as infected immediately upon detection, and corresponding movement restrictions were applied from the subsequent weekly time step. Thus, higher values of *α* proportionally reduced spatial dispersal and constrained long‐range spread originating from newly infected patches.

The intervention scenarios were applied across all stochastic simulation runs for each outbreak period, maintaining the same initial seeding and seasonal transmission parameters. By comparing intervention scenarios with the baseline (no restriction), we quantified reductions in high‐risk patches, shifts in regional outbreak likelihood, and heterogeneity in intervention responsiveness across provinces.

This framework integrates fence‐like physical containment and reactive quarantine measures into a unified stochastic modeling framework. By evaluating the impact of movement restrictions on ASF spatial dynamics, this approach provides valuable insights for supporting policy decisions and practical disease management tailored to Korea’s epidemiological and ecological contexts.

## 3. Results

### 3.1. Seasonal Transmission Pattern and Parameter Estimation

**Figure 5:**
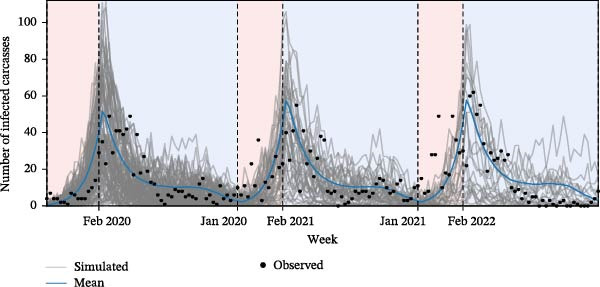
Parameter estimation results. Stochastic simulated epidemic curve (gray‐colored line), mean simulation trajectory (blue‐colored line), and observed number of ASF‐infected carcasses (dots). Vertical dashed lines indicate the transition points between the increase and decrease phases. Red‐ and blue‐shaded areas represent the increase and decrease phases, respectively.

We estimated seasonally varying transmission parameters as described in Section 2.2.3 by fitting the deterministic SICR model to weekly carcass surveillance data from October 2019 to September 2022. Across the three calibrated annual cycles, the estimated duration of the increase phase (*ω*) was 13 weeks, spanning roughly the November–January period. The turning point (*t*_0_) occurred around Week 16, after which the seasonal transmission pattern evolves periodically with a 52‐week cycle (Figure 5). The resulting transmission parameters differed markedly between phases, with the effective reproduction number *R*_*t*_ averaging 1.33 during increase phases and 0.89 during decrease phases (Table S4).

We also incorporated a seasonally varying carcass infectious period (1/*δ*_*c*_(*t*)). Among candidate specifications, the combination corresponding to infectious periods of 37 days under colder conditions and 8 days under warmer conditions showed the closest agreement with the observed carcass counts (RMSE = 11.081; Figure S1 and Table S5).

### 3.2. Spatial Spread Simulation and Region‐Specific Risk Assessment

Using the estimated transmission parameters, we evaluated the spatial spread of ASF across Korea within a patch‐based stochastic framework. The spatial configuration of the model was informed by an SDM constructed from 15 environmental and landscape variables (Table S3).

**Figure 6:**
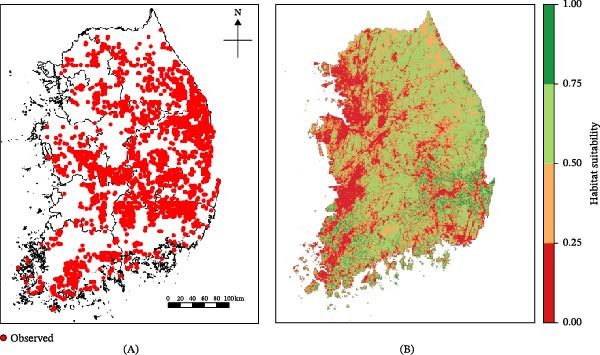
Estimation of wild boar habitat suitability using Maxent‐based SDM in Korea. (A) Locations of wild boar observation data from 2019 to 2023. (B) Predicted habitat suitability map generated using Maxent‐based SDM.

The SDM exhibited adequate overall discrimination, with an AUC of 0.741. Classification outcomes varied across thresholds, reflecting trade‐offs between sensitivity and specificity. At a low threshold (0.3), suitability was assigned broadly across patches, producing high sensitivity (96.6%) but low specificity (34.7%). Conversely, at a high threshold (0.8), suitability was restricted to a small subset of patches, resulting in substantially reduced sensitivity (2.6%) but high specificity (99.2%). The TSS reached its maximum value (0.3679) at a threshold of 0.5206, at which sensitivity and specificity were 85.6% and 51.2%, respectively (Figure 6 and Table S6).

**Figure 7:**
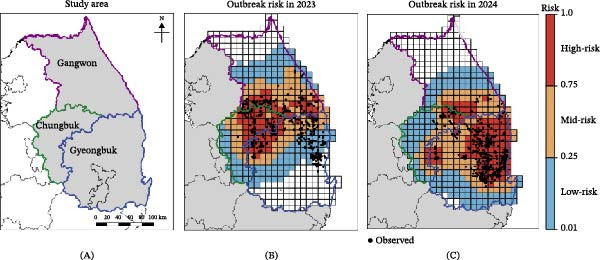
Spatial distribution of regional case coverage by year based on simulation results. (A) Marks the region names and locations, with areas outlined in purple, blue, and green corresponding to Gangwon, Gyeongbuk, and Chungbuk, respectively. Infected patches across Korea are classified into high‐ (red), mid‐ (orange), and low‐risk (blue) levels based on simulated outbreak probabilities for the 2023 (B) and 2024 (C) outbreaks.

Suitability probabilities estimated by the SDM were aggregated to the patch structure to derive movement probabilities, which were used to simulate ASF progression during the 2023 and 2024 outbreak periods. Figure 7 presents the spatial distribution of predicted outbreak risk and associated hot spots, representing regions with a high concentration of high‐risk patches. During the 2023 outbreak period, 81 patches were classified as high risk, compared with 84 high‐risk patches in 2024. The dominant hot spot shifted between years: Chungbuk accounted for the largest share of high‐risk patches in 2023 (33 of 89 patches, 40.74%), whereas Gyeongbuk emerged as the primary hot spot in 2024, with 76 of 222 patches (34.23%) classified as high risk. Together, these patterns indicate an expanded footprint consistent with the continued southward progression of ASF.

Table 2 summarizes case coverage by predicted risk category, indicating the degree of overlap between predicted risk areas and observed ASF detections. Nationally, 62.14% of 2023 cases and 81.43% of 2024 cases occurred within high‐risk patches (high‐risk coverage, Coverage_Korea, high_). When mid‐risk patches were additionally included (mid‐risk coverage, Coverage_Korea, mid_), coverage rose to 79.34% and 93.93%, respectively. Considering all of high‐, mid, and low‐risk patches (low‐risk coverage, Coverage_Korea, low_) yielded coverage above 96.8% in 2023 and reached 100% in 2024 [low‐risk coverage, Coverage_Korea, low_]) yielded coverage above 96.80% in 2023 and reached 100% in 2024.

**Table 2:** Coverage of reported ASF cases by model‐predicted risk levels.

Outbreak	Region	Coverage_*g*, high_(%)^a^	Coverage_*g*, mid_ (%)	Coverage_*g*, low_ (%)	Brier score
2023	Korea	62.14	79.34	93.93	0.1321
	Chungbuk	99.35	100	100	0.1634
	Gangwon	85.85	99.02	100	0.0788
	Gyeongbuk	30.12	57.53	87.35	0.1695

2024	Korea	81.43	95.39	96.80	0.1143
	Chungbuk	53.25	87.01	100	0.1389
	Gangwon	66.67	97.22	100	0.0838
	Gyeongbuk	91.92	100	100	0.1335

^a^Regional case coverage for each region (*g* ∈ {Chungbuk,  Gangwon,  Gyeongbuk,  Korea}) and risk level (level ∈ {low, ~ mid, ~ high}), defined in Section 2.3.3.

At the patch level, high‐risk classification yielded overall accuracies of 82.7% and 86.5% in 2023 and 2024, respectively (Table S7), whereas the proportion of patches classified as high risk in the absence of an observed outbreak remained low at 4.4% and 5.9% in 2023 and 2024, respectively. Accordingly, probabilistic predictive performance was supported by Brier scores of 0.1321 and 0.1143 in 2023 and 2024, respectively, indicating close concordance between predicted outbreak probabilities and the distribution of observed outbreak patches.

Although probabilistic predictive performance was adequate across regions (Brier scores, 0.0788–0.1695), regional differences were observed in case coverage across predicted risk categories. In 2023, Chungbuk and Gangwon exhibited high correspondence between predicted risk areas and observed outbreaks, with high‐ and mid‐risk coverages exceeding 85% and 99%, respectively. Conversely, Gyeongbuk demonstrated reduced coverage in 2023, with 30.12% high‐risk coverage and only 25.86% of outbreak patches classified as high risk (sensitivity: 15 of 58 observed outbreak patches). This discrepancy reflects the abrupt southward expansion that occurred during November–December 2023, when ASF simultaneously emerged across multiple distant patches, patterns not fully captured by gradual suitability‐driven dispersal (Figure S3E). In 2024, the majority of reported cases occurred in Gyeongbuk, with high‐risk coverage reaching 91.92%. Chungbuk and Gangwon exhibited lower high‐risk coverage during this period, consistent with a shift in predicted risk toward southeastern regions. Nevertheless, both provinces exceeded 87% coverage when mid‐risk patches were included, and all provinces reached full coverage at the low‐risk level. In addition, beyond these predefined risk categories, examination of case coverage across alternative risk thresholds revealed a consistent trend of increasing regional coverage as the threshold decreased (Table S8).

To assess the robustness of these spatial spread predictions, we conducted sensitivity analyses by varying key structural and parameter assumptions that influence the extent of predicted high‐risk areas (Table S9). The spatial locations of major high‐risk patches remained stable across these variations, with Chungbuk dominating in 2023 and Gyeongbuk in 2024 (Figure S2). Among structural assumptions, allowing longer‐range movement (permitting two‐patch movements) expanded the number of high‐risk patches from 81 to 112 in 2023 (+38.3%) and from 108 to 156 in 2024 (+44.4%). Conversely, reducing the patch size to 5 km × 5 km subsequently contracted high‐risk areas, with reductions of 87.7% and 78.5% in 2023 and 2024, respectively.

Among parameter variations, increasing the reporting delay (1/*δ*) produced the largest expansion of high‐risk areas, with the number of high‐risk patches rising to 122 (+50.6%) and 171 (+58.3%) in 2023 and 2024, respectively. Conversely, decreasing the long‐range dispersal proportion (*κ*) yielded the strongest contraction, reducing high‐risk patches to 39 (−51.9%) and 57 (−47.2%) in 2023 and 2024, respectively. Changes in detection rate (*f*) had comparatively modest effects, with reductions of 33.3% in 2023 and 25.0% in 2024 under lower detection and increases of ~8%–9% under intensified detection.

### 3.3. Effects of ASF Intervention Strategies

We evaluated two intervention strategies using the stochastic transmission model: (i) population reduction via intensified hunting and (ii) movement restriction implemented as reactive suppression of dispersal from infected patches.

Increasing hunting intensity was examined by raising the weekly hunting rate (hunting‐related surveillance parameter, *μ*_*p*_) up to threefold relative to its baseline value (0.0135/week; Table S1). As shown in Figure S4, higher hunting intensity was associated with a gradual reduction in the proportion of high‐risk patches across Korea, decreasing from 0.171 to 0.135 and from 0.227 to 0.211 during the 2023 and 2024 outbreak periods, respectively.

In contrast, movement restriction led to substantially larger reductions in the extent of high‐risk areas than population reduction alone, across both outbreak periods and all three restriction‐intensity scenarios (*α* = 0.3, 0.5, and 0.8; Table 3). During the 2023 outbreak period, the number of high‐risk patches decreased from 81 under baseline conditions (no restriction) to 52 (−35.8%), 39 (−51.9%), and 31 (−61.7%) as restriction intensity increased, with the corresponding proportion decreasing from ~0.171 to 0.065. A similar pattern was observed in 2024, with high‐risk patches declining from 108 at baseline to 77 (−28.7%), 61 (−43.5%), and 45 (−58.3%), and the corresponding proportion decreasing from 0.227 to 0.095.

**Table 3:** High‐risk infected‐patches counts and reduction rates by region and restriction intensity.

Outbreak	Region	Baseline (*α* = 0)	*α* = 0.3^a^	*α* = 0.5	*α* = 0.8
2023	Korea	81	52 (−35.8%)	39 (−51.9%)	31 (−61.7%)
	Chungbuk	33	21 (−36.4%)	13 (−60.6%)	10 (−69.7%)
	Gangwon	28	17 (−39.3%)	15 (−46.4%)	12 (−57.1%)
	Gyeongbuk	20	14 (−30.0%)	11 (−45.0%)	9 (−55.0%)

2024	Korea	108	77 (−28.7%)	61 (−43.5%)	45 (−58.3%)
	Chungbuk	11	7 (−36.4%)	3 (−72.7%)	2 (−81.8%)
	Gangwon	13	8 (−38.4%)	5 (−61.5%)	4 (−69.2%)
	Gyeongbuk	84	62 (−26.2%)	53 (−36.9%)	39 (−53.6%)

^a^Number of infected patches classified as high risk (reduction rate compared to baseline).

The spatial patterns of these reductions indicate that suppressing dispersal from newly infected patches effectively limits downstream spread along movement pathways shaped by habitat suitability. The response to movement restriction varied across regions (Figure 8 and Figure S5). During the 2023 outbreak, Chungbuk exhibited the largest outbreak observations, with 33 high‐risk patches and corresponding to a regional proportion of 0.434. Under the highest restriction level (*α* = 0.8), this decreased to 10 high‐risk patches (−69.7%), and the corresponding proportion declined to 0.132. Gangwon, despite a similar baseline number of high‐risk patches (28), showed the most responsive pattern under the weaker restriction level (*α* = 0.3). Gyeongbuk, which initially had fewer high‐risk patches (20), experienced smaller absolute reductions even though proportional decreases were comparable (−55.0% under *α* = 0.8). During the 2024 outbreak period, Gyeongbuk accounted for the majority of high‐risk patches at baseline (84 of 108) and corresponding to a regional proportion of 0.404. Under the highest restriction level (*α* = 0.8), this proportion decreased to 0.188, accounting for a 53.6% reduction. Chungbuk showed a pronounced proportional decline (−81.8% under *α* = 0.8), with the number of high‐risk patches decreasing from seven to two, whereas Gangwon remained stable, with a single high‐risk patch throughout the period.

Figure 8Effect of restriction intensity (*α*) on the number of high‐risk infected patches (2023 and 2024). (A–D) Spatial distribution of infected patches under different restriction intensity for 2023, (E–H) for 2024. Areas outlined in purple, blue, and green correspond to Gangwon, Gyeongbuk, and Chungbuk, respectively, while (A) additionally marks the region names and locations.

**Figure:**
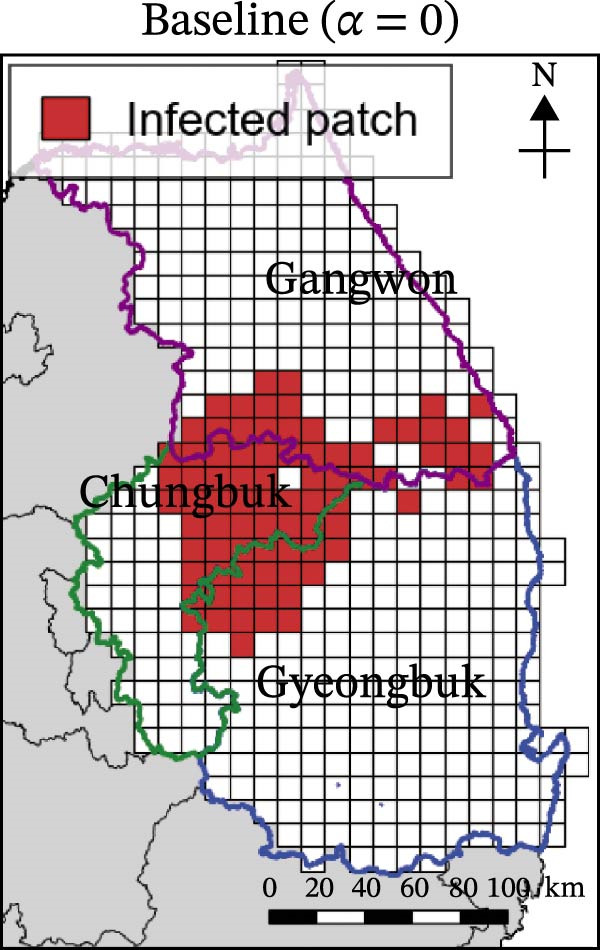
(A)

**Figure:**
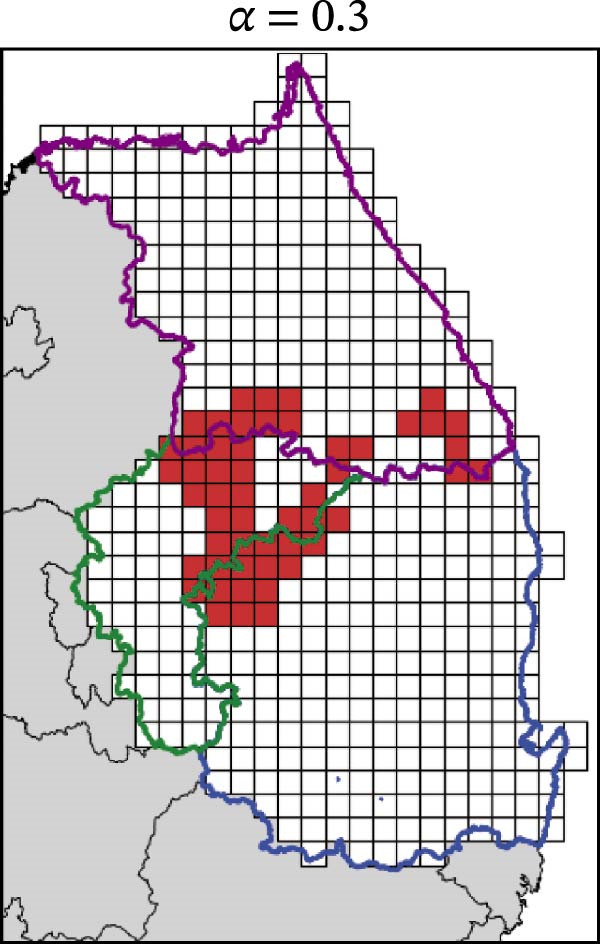
(B)

**Figure:**
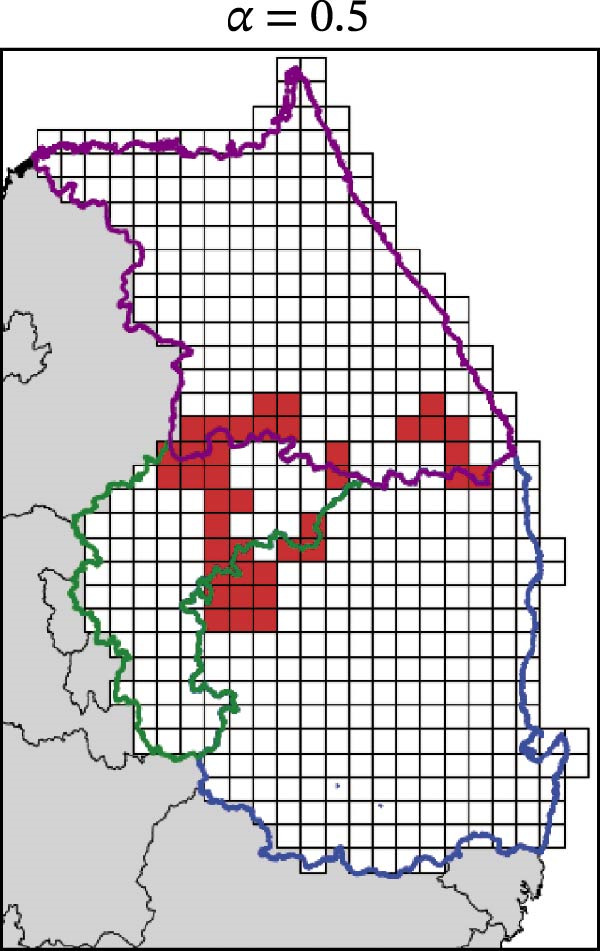
(C)

**Figure:**
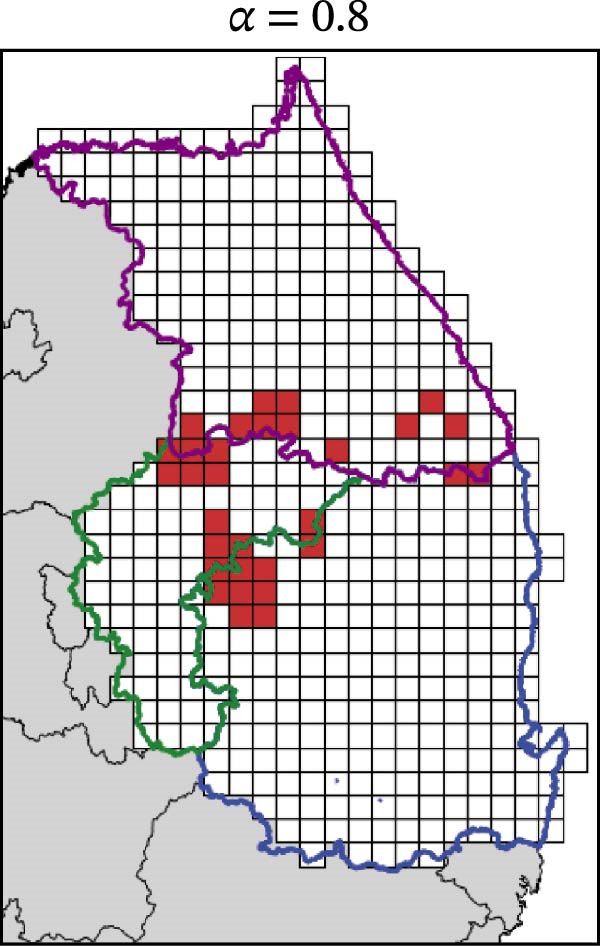
(D)

**Figure:**
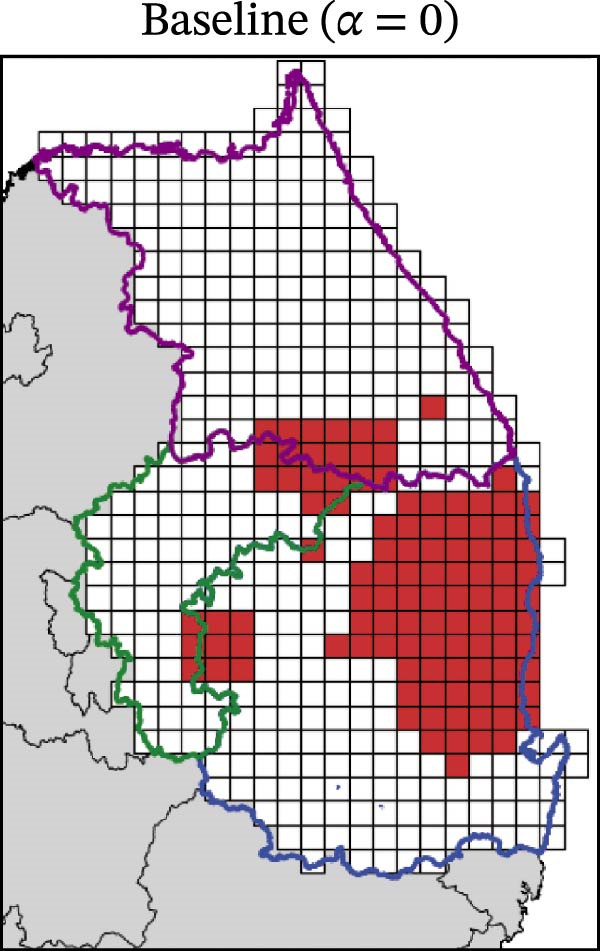
(E)

**Figure:**
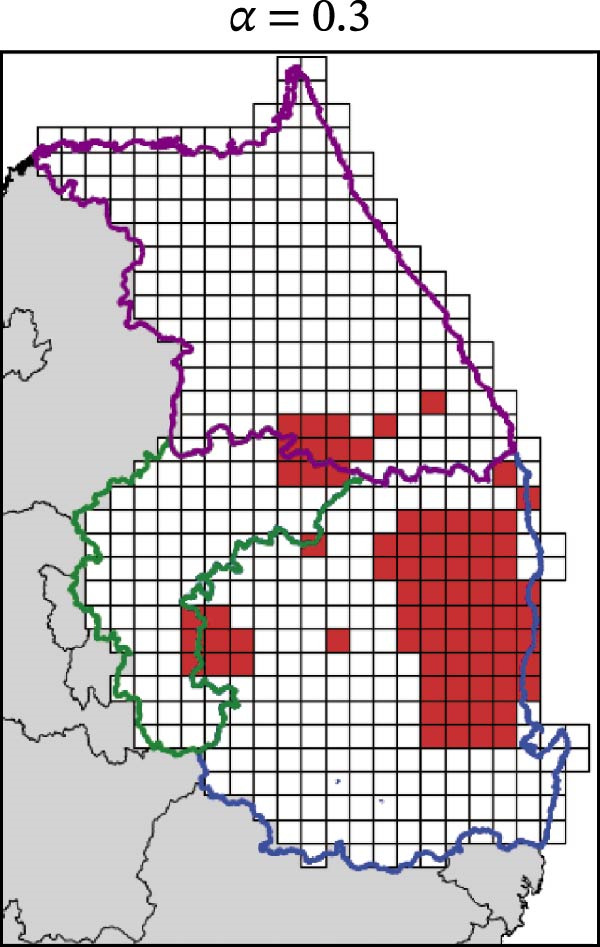
(F)

**Figure:**
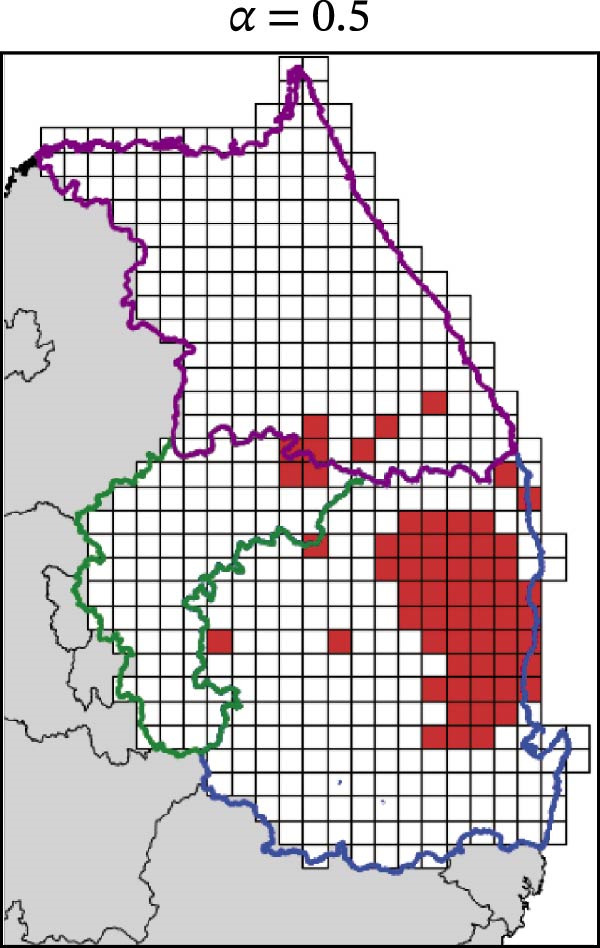
(G)

**Figure:**
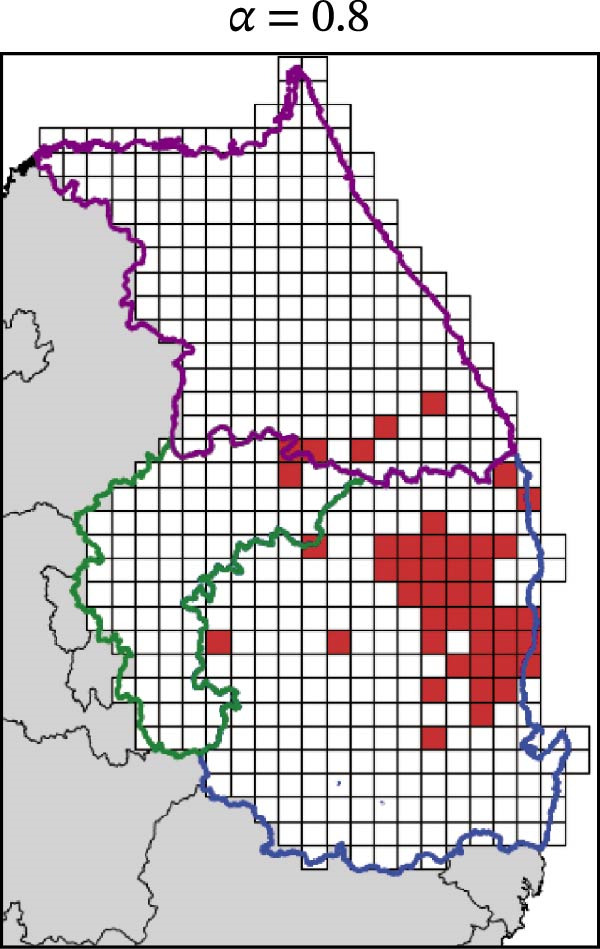
(H)

Overall, population reduction via intensified hunting produced only limited reductions in the spatial extent of ASF risk. By contrast, movement restriction consistently achieved substantially larger and more spatially structured reductions in high‐risk areas across outbreak periods. The extent and spatial patterns of these reductions varied by region, suggesting the potential role of spatially targeted mobility suppression in ASF control strategies.

## 4. Discussion

In this study, we developed a patch‐based stochastic model to explore wild boar–driven ASF spread by integrating habitat suitability into spatial movement probabilities. We quantified how infection intensity and spatial propagation evolve over time, revealing ecological dynamics that sustain persistent ASF epidemics in Korea and informing the evaluation of wildlife‐focused control strategies.

The model reproduced major features of the 2023–2024 outbreaks with high spatial correspondence, including shifts in dominant high‐risk regions from Chungbuk in 2023 to Gyeongbuk in 2024. Across both outbreak periods, classification for high‐risk areas exceeded 80%, and probabilistic performance was supported by Brier scores below 0.17, indicating close agreement between predicted outbreak probabilities and observed detections. Seasonal transmission dynamics were also well captured, with elevated *R*_*t*_ values during November–January averaging ~1.33, followed by a decline phase during the subsequent months with mean *R*_*t*_ ~0.89. These patterns align with ecological features, including intensified wild boar movement during the breeding season and prolonged carcass infectivity in cold environments [18, 57, 58]. Regionally, predicted risk patterns aligned closely with observed outbreaks in Chungbuk and Gangwon during the 2023 outbreak, where mid‐risk coverage exceeded 99% (Figure 7). In contrast, Gyeongbuk exhibited lower correspondence that year (30.12% of detections in high‐risk patches and 87.35% in any risk level), reflecting the abrupt and geographically dispersed emergence observed between September and December. Such patterns are unlikely to arise solely from stepwise suitability‐driven dispersal and may involve human‐mediated or rare long‐distance movements. In 2024, as outbreaks became concentrated in southeastern regions, the model captured 91.92% of reported Gyeongbuk detections within high‐risk patches. Intervention analyses further indicated that reactive movement restriction triggered by carcass detection substantially reduced the spatial extent of predicted high‐risk areas (−61.7% in 2023 and −58.3% in 2024 under *α* = 0.8). Sensitivity analyses showed that reporting delay and long‐range dispersal primarily affected the extent of predicted high‐risk areas (+51%–58% and −47%–52%, respectively), while the locations of dominant hot spots remained stable, centered in Chungbuk in 2023 and Gyeongbuk in 2024.

Previous ASF modeling studies have often focused on specific components of disease dynamics. For example, Taylor et al. [43] developed a spatially explicit ASF model in which wild boar movement and spatial distribution structured the spread of infection, whereas Dankwa et al. [44] examined distance‐based dispersal within a wild boar–domestic pig system using synthetic data. Han et al. [74] further proposed a simulation framework to estimate transmission parameters in Korea. Our model extends these approaches by integrating habitat‐informed dispersal probabilities, seasonally varying transmission dynamics, and carcass‐persistence effects within an SICR stochastic model, calibrated using empirical ASF surveillance data. This integration enables more realistic representation of observed spatial dynamics and provides a mechanistic explanation for outbreak progression across heterogeneous landscapes.

Beyond methodological advances, several empirical studies of Korean ASF outbreaks have evaluated interventions, such as fencing, using observed case distributions. Fencing, one of the major control interventions in Korea, has often shown modest or inconsistent effects [3, 75–77]. These outcomes likely reflect practical constraints, such as fences being installed along predicted movement routes [34, 77], as well as confounding influences from hunting pressure or food availability [47], which can obscure the intended impact of movement barriers. Acknowledging these constraints, our model implements fencing through its functional role as movement suppression rather than as explicit linear barriers, enabling fine‐scale evaluation of fence‐like restrictions and supporting more flexible scenario‐based analyses.

This study had certain limitations. First, the model cannot fully capture abrupt, large‐scale expansions, such as those observed in Gyeongbuk during September–December 2023 (Figure S3E), which exceed the stepwise, adjacency‐based movement assumptions inherent to the patch structure. Nonetheless, high‐risk patches retained epidemiological relevance, with 75.0% experiencing observed outbreaks (precision: 15 of 20 patches). Expanding evaluation to include mid‐ and low‐risk patches increased spatial coverage to above 85%, supporting the model’s robustness for landscape‐level assessment. Second, the model does not explicitly incorporate the precise geographical layout of Korea’s fencing network, as detailed spatial data are not publicly available. Instead, fences were represented phenomenologically as localized movement suppression. Furthermore, as high‐resolution fence geospatial data become available, the framework could be refined to incorporate patch‐specific directional constraints, supporting fine‐scale optimization of fence placement in operational planning.

To the best of our knowledge, this is the first study to develop and apply a patch‐based stochastic model that integrates habitat suitability–informed wild boar movement and fence‐mediated movement suppression to assess ASF transmission dynamics using empirical surveillance data in Korea. In addition, the model enables patch‐level identification of outbreak hot spots and supports the design of spatially targeted, adaptive management strategies. This approach may also inform modeling of other wildlife‐associated diseases in which ecological connectivity or time‐varying transmission dynamics play substantial roles. Scenario analyses across varying restriction intensities further help clarify the relative effectiveness of interventions and provide evidence to support policy decisions tailored to Korea’s ecological and epidemiological contexts. Moreover, the patch‐based stochastic modeling framework developed in this study is readily applicable to geographical areas beyond Korea. The SICR‐based transmission structure can be adapted by modifying compartments to reflect region‐specific ecological characteristics and surveillance systems, while the spatial patch network can be reformulated by adjusting patch size and connectivity to match country‐specific landscape configurations and monitoring units. Furthermore, habitat suitability layers derived from additional environmental variables can be incorporated to capture wild boar movement under varying environmental conditions. The component that models the barrier effects induced by movement restrictions can be readily extended to support alternative ASF control strategies, thereby enabling patch‐level management planning (such as prioritizing high‐risk patches) and tailored scenario evaluations for diverse management objectives.

## 5. Conclusion

Overall, this study presents a novel patch‐based stochastic modeling framework that explicitly incorporates habitat‐driven wild boar movement, underscoring its important role in ASF transmission dynamics. By discretizing the Korean landscape into uniform spatial units, the framework enables spatial risk assessment and facilitates the identification of localized outbreak hot spots. It provides a robust foundation for designing and evaluating spatially targeted intervention strategies at much higher spatial resolutions, thereby enhancing the effectiveness and efficiency of disease control efforts.

## Author Contributions

**Changdae Son:** data curation, visualization, methodology, formal analysis, writing – original draft, writing – review and editing. **Yongin Choi:** methodology, formal analysis, writing – original draft, writing – review and editing. **Hyojung Lee:** methodology, visualization, supervision, writing – original draft, writing – review and editing.

## Funding

Hyojung Lee was supported by the National Research Foundation of Korea (NRF) grant funded by the Korean Government (MSIT) (Grants RS‐2022‐NR070839 and NRF‐2022R1C1C1006237). Yongin Choi was supported by the National Research Foundation of Korea (NRF) grant funded by the Korean Government (MSIT) (Grant RS‐2024‐00407300). This research was supported by a grant for the project for the Government‐wide R&D to Advance Infectious Disease Prevention and Control, Republic of Korea (Grant HG23C1629) and the Basic Science Research Program through the National Research Foundation of Korea (NRF) funded by the Ministry of Education (Grant RS‐2024‐00466140).

## Conflicts of Interest

The author declare no conflicts of interest.

## Supporting Information

Additional supporting information can be found online in the Supporting Information section.

## Supporting information


**Supporting Information** Appendix A. Epidemic model. Appendix A.1: Seasonality for infectivity loss period of an infected carcass. Appendix A.2: Formulation of the effective reproduction number through the next‐generation matrix method. Appendix A.3: Specifications of initial conditions. Appendix B. Detailed description of the species distribution model. Appendix B.1: Species distribution modeling variables. Appendix B.2: Species distribution model. Table S1: Parameter description. Table S2: Notation for the patch‐based stochastic movement model. Table S3: Input variables for SDM. Table S4: Estimated transmission rates for ASF increase and decrease phases. Table S5: Comparison of candidate seasonal infectiousness‐loss periods. Table S6: Comparison of SDM performance metrics with different thresholds. Table S7: Patch‐level confusion matrices. Table S8: Coverage results under alternative outbreak‐probability thresholds. Table S9: Summary for sensitivity analyses. Figure S1: Weekly ASF‐infected carcass counts from 2019 to 2024, compared with simulated carcass trajectories generated using the seasonally varying carcass infectiousness‐loss rate. Figure S2: ASF outbreak risk using alternative movement‐range and patch‐size settings. Figure S3: Spatiotemporal distribution of ASF‐infected carcasses from 2019 to 2024. Figure S4: Proportion of infected patched by region according to hunting rate. Figure S5: Proportion of infected patches by region according to restriction intensity.

## Data Availability

The summary of the data is included in Table S3, Figures S1 and S3, and Figures 2, 5–7. Further inquiries can be directed to the corresponding author.
