# Supplementary material for: Modeling the Spatiotemporal Spread and Control of African Swine Fever in the Republic of Korea Using a Patch‐Based Stochastic Framework
Source: Transbound Emerg Dis. 2026 May 25;2026:9997936. doi: 10.1155/tbed/9997936 (PMC13199858; doi:10.1155/tbed/9997936)
Supplement: Supplementary file 1 — Supporting Information Appendix A. Epidemic model. Appendix A.1: Seasonality for infectivity loss period of an infected carcass. Appendix A.2: Formulation of the effective reproduction number through the next‐generation matrix method. Appendix A.3: Specifications of initial conditions. Appendix B. Detailed description of the species distribution model. Appendix B.1: Species distribution modeling variables. Appendix B.2: Species distribution model. Table S1: Parameter description. Table S2: Notation for the patch‐based stochastic movement model. Table S3: Input variables for SDM. Table S4: Estimated transmission rates for ASF increase and decrease phases. Table S5: Comparison of candidate seasonal infectiousness‐loss periods. Table S6: Comparison of SDM performance metrics with different thresholds. Table S7: Patch‐level confusion matrices. Table S8: Coverage results under alternative outbreak‐probability thresholds. Table S9: Summary for sensitivity analyses. Figure S1: Weekly ASF‐infected carcass counts from 2019 to 2024, compared with simulated carcass trajectories generated using the seasonally varying carcass infectiousness‐loss rate. Figure S2: ASF outbreak risk using alternative movement‐range and patch‐size settings. Figure S3: Spatiotemporal distribution of ASF‐infected carcasses from 2019 to 2024. Figure S4: Proportion of infected patched by region according to hunting rate. Figure S5: Proportion of infected patches by region according to restriction intensity. [file TBED-2026-9997936-s001.docx]

**Appendix A. Epidemic model**

**A.1 Seasonality for infectivity loss period of an infected carcass**

The infectivity loss period of ASF-infected carcasses varies seasonally because carcass decomposition is influenced by environmental conditions, particularly temperature [1, 2]. Infectivity tends to persist longer under cold conditions, whereas warmer temperatures accelerate decomposition and shorten this period. To represent this seasonal pattern, we modeled the carcass infectiousness-loss rate $\delta_{c}(t)$ using the periodic formulation proposed by Shaw et al. (2024) [3]:

$\delta_{c}\left( t \right)=\delta_{0, c}exp(-s\left( \frac{\pi\left( t-\Phi\right)}{T} \right) )$,

where $T$ denotes the annual period, $\delta_{0, c}$ is the baseline loss rate, $s$ is the seasonal amplitude, and $\Phi$ is the phase parameter.

In this study, we set $T=52$ weeks, consistent with the weekly simulation step and the annual recurrence of carcass infectivity, which is prolonged during colder seasons (fall$-$winter) and shortened during warmer seasons (spring$-$summer). The phase parameter $\Phi$ was chosen so that $\delta_{c}\left( t \right)$ achieves its minimum (i.e., longest infectious duration) in the middle of the colder season and its maximum (i.e., shortest infectious duration) in the middle of the warmer season. We assume these points correspond to epidemiological week 48 (mid-cold season) and week 22 (mid-warm season), respectively. The parameters $\delta_{0, c}$ and $s$ were then selected so that the periodic function matched the intended maximum and minimum infectious periods at these seasonal midpoints.

As candidate seasonal ranges, we considered three maximum-minimum pairs—(365/7 weeks, 90/7 weeks), (30/7 weeks, 7/7 weeks), (37/7 weeks, 8/7 weeks)—as reported in Fisher et al. (2020) and Probst et al. (2019) [1, 2]. A periodic function was constructed for each parameter set. For every candidate function, we estimated the transmission rate $\beta(t)$ and evaluated model performance by comparing simulated and observed ASF-infected carcass counts using the root-mean-square error (RMSE) (Supplementary Figure S1 and Table S4). The seasonal specification that produced the lowest RMSE was selected as the final model for representing carcass infectivity dynamics.

**A.2 Formulation of the effective reproduction number through the next-generation matrix method.**

The effective reproduction number ($R_{t}$) was derived from using the next-generation matrix (NGM) method [4, 5]. The compartmental model described in Section 2.2.2 consists of four compartments, namely susceptible (*S*), infectious (*I*), carcass (*C*), and reported (*R*), represented by the vector $x=\left( S, I, C, R \right)^{T}$.

To apply the NGM method, we focused on the infectious compartments that contribute to the generation of new infections, namely $I$ and $D$. The infection process was conceptually divided into two components. The first is $F(x)$, which represents the rate at which new infections appear. The second is $V(x)$, which describes the rate of transfer among infected compartments, including processes such as detection, and removal.

Let $x=\left( I, C \right)^{T}$. Based on the dynamics of models, the functions $F(x)$ and $V(x)$ are given by

$F\left( x \right)=\left[ \beta(I+\theta C) 0 \right], V\left( x \right)=\left[ \left( \gamma+\mu+\mu_{p} \right)I \gamma I+(f\delta+\left( 1-f \right)\delta_{c})C \right]$.

At the disease-free equilibrium, we computed the Jacobian matrices of $F(x)$ and $V(x)$ with respect to $x$, resulting in the transmission matrix $F$ and the transition matrix $V$:

$F=\left[ \beta\beta\theta0 0 \right], V=\left[ f\delta+\left( 1-f \right)\delta_{c} 0 \gamma\gamma+\mu+\mu_{p} \right]$.

The inverse of $V$ was then calculated as

$V^{-1}=\left[ \frac{1}{\gamma+\mu+\mu_{p}} 0 \frac{\gamma}{(\gamma+\mu)(f\delta+\left( 1-f \right)\delta_{c})} f\delta+\left( 1-f \right)\delta_{c} \right]$

Using this, we obtained the next-generation matrix $K$ as

$K=FV^{-1}=\left[ \frac{\beta}{\gamma+\mu+\mu_{p}}+\frac{\beta\theta}{f\delta+\left( 1-f \right)\delta} \frac{\beta\theta}{f\delta+\left( 1-f \right)\delta_{c}} 0 0 \right]$.

The basic reproduction number was first computed as the spectral radius of the next-generation matrix,

$R=\rho\left( K \right)=\frac{\beta}{\gamma+\mu+\mu_{p}}+\frac{\beta\theta}{f\delta+\left( 1-f \right)\delta_{c}}$,

where $\rho\left( \cdot\right)$ denotes the dominant eigenvalue. The effective reproduction number at time $t$, denotes $R_{t}$, was then obtained by adjusting the proportion of susceptible individuals,

$R_{t}=\frac{S\left( t \right)}{Pop\left( t \right)}\left( \frac{\beta\left( t \right)}{\gamma+\mu+\mu_{p}}+\frac{\beta\left( t \right)\theta}{f\delta+\left( 1-f \right)\delta_{c}} \right)$.

Reflecting both temporal changes in the transmission rate $\beta(t)$ and the number of susceptible $S\left( t \right)$ may differ from the total population $Pop\left( t \right)$ over time.

**A.3 Specification of initial conditions**

Initial conditions were defined using reported carcass data and wild-boar surveillance data available at the start of the simulation. Reported carcasses were used to infer initial epidemiological states while accounting for reporting delay inherent to carcass-based surveillance, and surveillance data were used to determine population size and spatial distribution.

**Population scaling**

The total wild-boar population was scaled using monitoring data from the National Institute of Ecology to match an expected mean density of 2.3 boars per $km^{2}$ over a national land area of 99,678 $km^{2}$ [6]. This scaling yields an estimated national wild-boar population of approximately 229,259 individuals (2.3 boars$\cdot km^{-2}$ $\times$ 99,678 $km^{2}$). Based on this scaling, the initial active wild-boar population across Korea was defined as $Pop(0)=229,259$.

**Initialization of the epidemiological states**

- **Infectious carcass state.** The initial number of infectious carcasses, $C(0)$, was defined by accounting for reporting delay in carcass-based surveillance [31]. At the start of the simulation (simulation time $t=0$), the reporting delay time was assumed to be approximately five weeks (37 days), with a reporting rate of $f=0.25$. Under this assumption, carcasses reported during the first five weeks of the simulation were treated as originating from carcasses that existed prior to $t=0$, with only a subset of existing carcasses being detected and reported [7, 34]. Let $Obs(t)$ denote the number of carcasses reported at simulation time $t$. The initial carcass count was then defined as

$C(0)=4\times\sum_{t=1}^{5} Obs(t)$.

- **Infected wild boar state.** Under the transition structure of the epidemic model, infected wild boars transition to the infectious carcass state at rate $\gamma=1$. To preserve this structural relationship between infected individuals and carcass accumulation [8], the initial number of infected wild boars ($I(0)$) was set equal to the initial carcass count,

$I(0)=C(0)$.

In addition, the reported carcass state was initialized as $R(0)=0$, and the susceptible population was defined as the remaining active population, $S(0)=Pop(0)-I(0)$.

**Patch-level Initialization for the stochastic model with movement**

Patch-level initial conditions were specified using patch-specific population and reported carcass data. The national wild-boar population was scaled from a directly observed count of 8,470 individuals to 229,259 individuals. The unobserved remainder (220,789 individuals) was allocated across patches in proportion to habitat suitability. The resulting initial population size for patch $i$ is denoted as $Pop_{i}(0)$ and was generated as follows:

$(Pop_{1}(0), Pop_{2}(0), ..., Pop_{N}(0)) \sim Multinomial(220,789, H(1), H(2), ..., H(W))$,

where $H(i)$ represents the habitat suitability of patch $i$, and $W=475$ denotes the total number of patches across Korea.

Let $Obs_{i}(t)$ denote the number of carcasses reported in patch $i$ at simulation time $t$. Patch-level epidemiological states were initialized as

$C_{i}(0)=4\times\sum_{t=1}^{5} Obs_{i}(t)$, $I_{i}(0)=C_{i}(0)$, $R_{i}(0)=0$, and $S_{i}(0)=Pop_{i}(0)-I_{i}(0)$.

**Appendix B. Detailed description of the species distribution model**

**B.1 Species distribution modeling variables**

**Wild-boar observation data**

In this study, we used wild boar observation data collected by the National Institute of Ecology [9] from 2019 to 2023 to estimate wild boar habitats. The dataset includes all observations of wild boars, both alive and dead, and consists of geographic coordinates (latitude and longitude) and observation periods. Considering that the survey regions varied across different time periods, only the coordinates of observation locations were used in the analysis to ensure consistency across time periods. In addition, we used wild boar habitat density and hunting data provided by the Ministry of Environment [10, 11]. The habitat density data were collected from 2019 to 2022. We used the wild boar habitat density surveyed in sample areas as the national-level habitat density and assumed that all regions in the Republic of Korea shared the same habitat density, regardless of regional environmental differences.

**Environmental and landscape data and input variables of the SDM**

To comprehensively assess the factors influencing wild boar habitats and ASF transmission, we incorporated topographic, climatic, socio-environmental, and ecological variables into the modeling framework, summarized in Table S3 in the supplementary materials and illustrated in Figure 2B and 2C.

Each data set was originally structured as spatial data with values defined for uniform-sized cells, with cell sizes specific to each dataset. Topological variables, including *Elevation, Slope Angle* and *Slope Aspect angle,* were derived from a Digital Elevation Model (DEM) provided by the National Geographic Information Institute [12]. The DEM is composed of a uniform 5m $\times$ 5m cells, each representing the elevation value at that cell. This elevation value was used as the *Elevation* variables in our analysis. Based on this elevation data, *Slope Angle* and *Slope Aspect angle* were calculated using Slope and Aspect tools in QGIS, which analyzed elevation differences between neighboring 5m $\times$ 5m cells in the DEM.

We also incorporated annual mean temperature, minimum temperature, maximum temperature and mean precipitation, sourced from the Korea Meteorological Administration (KMA) [13] to account for climatic variability affecting wild boar activity. The climate data consisted of annual records from 2019 to 2023, measured at multiple observation stations. To estimate values across Korea, we imported a shapefile of Korea, which follows the EPSG:5179 coordinate reference system and represents the national boundary as of July 2023. The area was divided into uniform 1km $\times$ 1km grid cells and inverse distance weighting interpolation was applied in QGIS to estimate climate values for each cell. This method was chosen since it has been used by the KMA and several previous studies [14, 15]. Additionally, we utilized land cover data from European Space Agency worldcover [16] to derive both ecological and human - related variables. Forest and river layers represent ecological features that provide essential resources for wild boars, such as food availability and access to drinking water. Cropland and building layers reflect human-modified landscapes that may influence wild boar habitats. These layers originally consisted of presence or absence data for specific landscape features in each uniform 10m $\times$ 10m cell. For analysis, we transformed these data into distance-based variables by calculating the distance from each cell to the nearest landscape element using Euclidean distance. In addition, we considered human-related variables of the *population density* by using the population density data from Worldpop [17], which are defined for uniform 1km $\times$ 1km cells. This variable was used to evaluate the potential impact of human presence on wild boar habitats.

All datasets were resampled to a common structure of uniform 1km $\times$ 1km cells for analysis, based on the coarsest resolution among the variables. By combining these topographic, climatic, ecological, and human-related variables, we constructed a comprehensive environmental dataset to support wild boar habitat suitability modeling and ASF transmission simulations in this study.

**B.2 Species distribution model**

To construct a spatially explicit ASF transmission model, we first quantified the relative habitat suitability for wild boars across Korea using a species distribution model (SDM) based on the maximum entropy (Maxent) algorithm. SDMs are widely utilized in ecological studies to predict species distributions from environmental variables [18–20]. Maxent derives a probability distribution consistent with environmental conditions observed at known occurrence locations. Maxent estimates habitat suitability by deriving a probability distribution that reflects environmental conditions observed at known occurrence locations, making it well suited for presence-only data and complex species–environment relationships [21–22].

Formally, let $X$ denote the set of all 1km $\times$ 1km grid cells across Korea, and $f_{j}(x)$ represent the value of the $j$-th environmental variable at location $x$. A total of 15 environmental predictors capturing topographic, climatic, ecological, and human-related factors were considered. The Maxent algorithm estimates the habitat suitability probability $p(x)$ of wild boars at location x by maximizing entropy, $H\left( p \right)=-\sum_{x\in X} p\left( x \right) log(p\left( x \right))$, subject to the constraint $\sum_{x\in X} p\left( x \right)f_{j}\left( x \right)=\bar{f_{j}}$, where $\bar{f_{j}}$ is its empirical mean of the $j$-th environmental variable across wild boar occurrence sites. This constraint ensures that model-predicted environmental features match observed conditions in expectation at occurrence sites. Solving this constrained optimization yields $p\left( x \right)=\frac{1}{Z}exp(\sum_{j} \lambda_{j}f_{j}(x))$, where $\lambda_{j}$ are coefficients associated with the environmental variables and $Z$ is a normalization constant ensuring $\sum_{x\in X} p(x)=1$.

Because explicit absence data were unavailable, we adopted a pseudo-absence strategy in which locations assumed to represent non-occurrence were included to support model fitting. Pseudo-absence points were randomly sampled across Korea at a ratio of three pseudo-absence points per presence record (1:3) [23–25].

The predictive performance of the SDM was evaluated using multiple complementary discrimination metrics to assess its suitability as an input risk structure for the ASF spatial transmission model [18, 26, 27]. Specifically, the following metrics were used:

- **Area under the receiver operating characteristic curve (AUC).** AUC measures the overall ability of the SDM to discriminate between wild boar presence and non-presence across all classification thresholds. As a threshold-independent metric, it provides a global assessment of model discrimination performance.
- **Sensitivity.** Sensitivity represents the proportion of observed wild boar occurrence locations correctly classified as suitable, indicating the model’s ability to capture areas potentially relevant for wild boar movement and ASF transmission.
- **Specificity.** Specificity represents the proportion of non-occurrence (pseudo-absence) locations correctly classified as unsuitable, reflecting the models’ ability to exclude areas with a low likelihood of wild boar use.
- **True Skill Statistics (TSS).** TSS combines sensitivity and specificity into a single balanced metric, $TSS=Sensitivity+Specificity-1$, and is robust to class imbalance, jointly accounting for errors arising from both missed presence locations and incorrectly included non-occurrence areas.

**Supplementary Table S1. Parameter description.**

| Parameter | Description | Value | Reference |
| --- | --- | --- | --- |
| $\beta(t)$ | Transmission rate at time $t$ | Estimated | - |
| $b$ | Wild boar birth rate ($week$) | 0.0133^1)^ | [28] |
| $\mu_{p}$ | Mortality rate due to wildlife population control measures ($week$) | 0.0135^2)^ | [29] |
| $\mu$ | Natural mortality rate of wild boar | 0.0028^3)^ | [30] |
| $1/\gamma$ | Infectious period for wild boars ($week$) | 1 | [31] |
| $1/\delta_{c}(t)$ | Infectivity loss period of an infected carcass $c$ at time $t$ ($week$) | $\frac{1}{\delta_{0, c}exp(-s cos^{2}(\pi(t-\phi)/T))}$ | Estimated |
| $1/\delta_{0,c}$ | Baseline loss period of $\delta_{c}\left( t \right)$ (week) | 8/7 | [2] |
| $s$ | Seasonal amplitude of $\delta_{c}\left( t \right)$ | 1.5315 | Estimated |
| $\phi$ | Phase of $\delta_{c}\left( t \right)$ | -18 | Assumed |
| $1/\delta$(t) | Reporting delay for detection and processing within the surveillance efforts ($week$) | $1/\delta_{c}(t)$ | Assumed |
| $f$ | Detection rate of infected carcasses | 0.25 | [31] |
| $\theta$ | Relative infectiousness of carcass–mediated transmission | 0.035 | [30] |
| $\kappa$ | Proportion of infectious individuals dispersing | 0.154 | [32] |

^1)^ The value is estimated as the product of 5.2 surviving offspring per litter, one litter per female per year, and a 50% female population share (per prior practice [3]), and then converted to a weekly basis.

^2)^ Annual hunting rate of approximately 70% reported in [10] was converted to a weekly basis.

^3)^ Annual mortality rate of 14.4% (from [29]) converted to a weekly basis.

**Supplementary Table S2. Notation for the patch-based stochastic movement model.**

| Symbol | Description |
| --- | --- |
| $i$, $j$ | Patch indices |
| $H(i)$ | Habitat suitability value of patch $i$ |
| $N(i)$ | Set of neighboring patches of patch $i$ |
| $P\left( i\to j^{(l)} \right)$ | Probability of wild boar movement from patch $i$ to an adjacent neighboring patch $j^{\left( l \right)}$, weighted by habitat suitability ($l=1, 2, ..., 8$) |
| $m_{i}\left( t \right)$ | Destination patch index assigned to infectious individuals moving outward from patch $i$ at time $t$ |
| $q_{j,i}\left( t \right)$ | Number of infectious individuals moving into patch $i$ from neighboring patch $j$ at time $t$ |
| $q_{i,m_{i}\left( t \right)}$^1)^ | Number of infectious individuals moving out of patch $i$ to destination patch patch $m_{i}(t)$ at time $t$ |

^1)^ Variables with an asterisk (*) indicate movement under policy-imposed restrictions.

**Supplementary Table S3. Input variables for SDM.**

| Category | Variable | Statistical summary^2)^ | Reference |
| --- | --- | --- | --- |
| Topographic variable | Elevation ($m$) | 291.52 (-1.97$-$1,614.56) | [12] |
|  | Slope Angle ($^{\circ}$) | 11.84 (0$-$41.39) |  |
|  | Slope Aspect Angle ($^{\circ}$) | 178.17 (0.08$-$360.0) |  |
| Climatic variable^1)^ | Annual mean temperature ($^{\circ}C$) | Year 2019: 13.12 (8.10$-$15.70) Year 2020: 12.90 (7.80$-$15.20)  Year 2021: 13.22 (7.90$-$15.80)  Year 2022: 12.83 (7.60$-$15.40)  Year 2023: 13.56 (8.50$-$15.40) | [13] |
|  | Annual maximum temperature ($^{\circ}C$) | Year 2019: 18.95 (13.60$-$21.30)  Year 2020: 18.43 (13.10$-$20.70)  Year 2021: 18.88 (13.30$-$21.40)  Year 2022: 18.63 (13.20$-$21.20)  Year 2023: 19.24 (14.00$-$21.70) |  |
|  | Annual minimum temperature ($^{\circ}C$) | Year 2019: 8.02 (2.70$-$12.50)  Year 2020: 8.13 (2.70$-$12.20)  Year 2021: 8.33 (2.90$-$12.80)  Year 2022: 7.73 (2.20$-$12.10)  Year 2023: 8.58 (3.20$-$12.80) |  |
|  | Annual mean precipitation ($mm$) | Year 2019: 1,155.84 (595.40$-$2,268.67)  Year 2020: 1,607.43 (1,128.91$-$2,471.78)  Year 2021: 1,218.77 (910.01$-$2,227.87)  Year 2022: 1,145.0 (581.00$-$2,143.38)  Year 2023: 1,700.61 (938.00$-$2,699.17) |  |
| Ecological variable | NDVI | 0.55 (-0.20$-$0.89) | [16] |
|  | Forest type^3)^ | 0 (of 30,416,406) and 1 (of 2,661,966) | [17] |
|  | Distance to river ($km$) | 2.13 (0$-$15.74) |  |
|  | Distance to forest ($km$) | 0.82 (0$-$21.03) |  |
| Human-related variable | Distance to cropland ($km$) | 0.65 (0$-$88.74) |  |
|  | Distance to road ($km$) | 2.31 (0$-$28.02) |  |
|  | Distance to building ($km$) | 0.79 (0$-$89.51) |  |
|  | Population density ($\left( km^{2} \right)^{-1}$) | 358.23 (0.18$-$25,024.07) | [33] |

^1)^ Climatic data were utilized for each year

^2)^ The statistical summary for numerical data provides the mean (range) of each variable used.

^3)^ Each value indicates the number of cells classified as 1 (broadleaf forest) or 0 (non-broadleaf forest).

**Supplementary Table S4. Estimated transmission rates for ASF increase and decrease phases.**

|  | Estimated period | Transmission rate ($\beta(t)$) | Mean of reproduction number^1)^ |
| --- | --- | --- | --- |
| **Increase phase** | Oct. 07, 2019 – Jan. 26, 2020 | $\beta_{increase}=$ 1.3055 | 1.3337 (1.3281-1.3435) |
|  | Nov. 01, 2020 – Jan. 31, 2021 |  | 1.3324 (1.3283-1.3408) |
|  | Nov. 01, 2021 – Jan. 30, 2022 |  | 1.3328 (1.3283-1.3436) |
| **Decrease phase** | Jan. 27, 2020 – Nov. 01, 2020 | $\beta_{decrease}=$ 0.8377 | 0.8966 (0.8399-0.9495) |
|  | Feb. 01, 2021 – Oct. 31, 2021 |  | 0.8925 (0.8386-0.9487) |
|  | Jan. 31, 2022 – Sep. 30, 2022 |  | - 1. (0.8386-0.9495) |

^1)^ Each value indicates the mean (95% credible interval) of $R_{t}$ for each period.

**Supplementary Table S5. Comparison of candidate seasonal infectiousness loss periods.**

| Maximum value of $1/\delta_{c}(t)$ | Minimum value of $1/\delta_{c}(t)$ | RMSE | Reference |  |
| --- | --- | --- | --- | --- |
| 365/7 | 90/7 | 12.584 | [1] |  |
| 30/7 | 7/7 | 13.055 |  |  |
| 37/7 | 8/7 | 11.081 | [2] |  |

**Supplementary Table S6. Comparison of SDM performance metrics with different thresholds.**

| Threshold | AUC | Sensitivity | Specificity | TSS |
| --- | --- | --- | --- | --- |
| 0.3000 | 0.7410 | **0.9660** | 0.3465 | 0.3124 |
| 0.8000 |  | 0.0264 | **0.9918** | 0.0182 |
| 0.5206^1)^ |  | 0.8559 | 0.5120 | **0.3679** |

^1)^ The threshold (0.5206) was selected as the value that maximized the TSS.

**Supplementary Table S7. Patch-level confusion matrices.**

| Threshold | Outbreaks | Region | Total patches^1)^ | TP^2)^ | FP^2)^ | TN^2)^ | FN^2)^ |
| --- | --- | --- | --- | --- | --- | --- | --- |
| 0.75 | 2023 | Korea | 475 | 60^3)^  (12.6%)^4)^ | 21  (4.4%) | 333  (70.1%) | 61  (12.8%) |
|  |  | Chungbuk | 76 | 23  (30.3%) | 10  (13.2%) | 42  (55.3%) | 1  (1.3%) |
|  |  | Gangwon | 191 | 22  (11.5%) | 6  (3.1%) | 146  (76.4%) | 17  (8.9%) |
|  |  | Gyeongbuk | 208 | 15  (7.2%) | 5  (2.4%) | 145  (69.7%) | 43  (20.7%) |
|  | 2024 | Korea | 475 | 80  (16.8%) | 28  (5.9%) | 331  (69.7%) | 36  (7.6%) |
|  |  | Chungbuk | 76 | 6  (7.9%) | 5  (6.6%) | 56  (73.7%) | 9  (11.8%) |
|  |  | Gangwon | 191 | 5  (2.6%) | 8  (4.2%) | 169  (88.5%) | 9  (4.7%) |
|  |  | Gyeongbuk | 208 | 69  (33.2%) | 15  (7.2%) | 106  (51.0%) | 18  (8.7%) |

^1)^ Number of patches belonging to the region

^2)^ TP (True positives): outbreak patches correctly classified as high risk; FP (False Positive): patches classified as high risk despite no observed outbreak; TN (True negatives): non-outbreak patches correctly classified as low risk; FN (False negatives): outbreak patches incorrectly classified as low risk

^3)^ Value indicates the number of patches classified into the corresponding category

^4)^ Value indicates the proportion of all patches in the region that belong to the corresponding category

**Supplementary Table S8. Coverage results under alternative outbreak-probability thresholds.**

| Outbreaks | Region | ${Coverage}_{\left( Region \right), risk threshold}(\%)$ | | | | | |
| --- | --- | --- | --- | --- | --- | --- | --- |
|  |  | 0.8^1)^ | 0.75^2)^ | 0.7 | 0.5 | 0.3 | 0.25^3)^ |
| 2023 | Korea | 59.68 | 62.14 | 65.03 | 72.98 | 77.31 | 79.34 |
|  | Chungbuk | 96.77 | 99.35 | 99.35 | 100 | 100 | 100 |
|  | Gangwon | 82.93 | 85.85 | 88.29 | 95.61 | 98.54 | 99.02 |
|  | Gyeongbuk | 28.01 | 30.12 | 34.64 | 46.39 | 53.61 | 57.53 |
| 2024 | Korea | 81.05 | 83.48 | 87.45 | 91.81 | 95.39 | 95.39 |
|  | Chungbuk | 50.65 | 53.25 | 62.34 | 63.64 | 87.01 | 87.01 |
|  | Gangwon | 63.89 | 66.67 | 66.67 | 83.33 | 97.22 | 97.22 |
|  | Gyeongbuk | 88.80 | 91.29 | 95.02 | 99.22 | 100 | 100 |

^1)^ Indices indicate the risk thresholds used in calculating the coverage of reported carcass locations based on predicted risk areas for each region.

^2)^ High ($Coverage_{g, high}(\%)$) was defined using an outbreak probability threshold of 0.75 ($\boldsymbol{p}_{\boldsymbol{i}}\boldsymbol{\geq0.75}$).

^3)^ Mid ($Coverage_{g, mid}(\%)$) was defined using an outbreak probability threshold of 0.25 ($\boldsymbol{p}_{\boldsymbol{i}}\boldsymbol{\geq0.25}$).

**Supplementary Table S9. Summary for sensitivity analyses.**

| Year | Region | Baseline^1)^ | Movement distance & Patch size | | $\kappa$ | | | $Pop(0)$ | | | $f$ | | | $1/\delta(t)$ | | |
| --- | --- | --- | --- | --- | --- | --- | --- | --- | --- | --- | --- | --- | --- | --- | --- | --- |
|  |  |  | Move^2)^ | Patch^3)^ | $\kappa\times0.5$ | $\kappa\times1.5$ | $Pop(0)\times0.5$ | | $Pop(0)\times1.5$ | $f\times0.5$ | | $f\times1.5$ | $1/\delta(t)\times0.5$ | | $1/\delta(t)\times1.5$ |  |
| 2023 | Korea | 81 | 112 (+38.3%)^4)^ | 10 (-87.7%) | 39 (-51.9%) | 118 (+45.7%) | 68 (-16.0%) | | 86 (+6.2%) | 54 (-33.3%) | | 88 (+8.6%) | 64 (-21.0%) | | 122 (+50.6%) |  |
|  | Chungbuk | 33 | 43 (+30.3%) | 3.25 (-90.2%) | 13 (-60.6%) | 41 (+24.2%) | 30 (-9.1%) | | 35 (+6.1%) | 24 (-27.3%) | | 35 (+6.1%) | 28 (-15.2%) | | 42 (+64.3%) |  |
|  | Gangwon | 28 | 40 (+42.9%) | 4.25 (-84.8%) | 15 (-46.4%) | 45 (+60.7%) | 22 (-21.4%) | | 30 (+7.1%) | 16 (-42.9%) | | 31 (+10.7%) | 20 (-28.6%) | | 46 (+64.3%) |  |
|  | Gyeongbuk | 20 | 29 (+45.0%) | 2.50 (-87.5%) | 11 (-45.0%) | 32 (+60.0%) | 16 (-20.0%) | | 21 (+5.0%) | 14 (-30.0%) | | 22 (+10.0%) | 16 (-20.0%) | | 34 (+70.0%) |  |
| 2024 | Korea | 108 | 156 (+44.4%) | 23.25 (-78.5%) | 57 (-47.2%) | 159 (+47.2%) | 90 (-16.7%) | | 111 (+2.8%) | 81 (-25.0%) | | 117 (+8.3%) | 87 (-19.4%) | | 171 (+58.3%) |  |
|  | Chungbuk | 11 | 15 (+36.4%) | 0.25 (-97.7%) | 3 (-72.7%) | 16 (+45.5%) | 7 (-36.4%) | | 12 (+9.1%) | 7 (-36.4%) | | 12 (+9.1%) | 7 (-36.4%) | | 19 (+72.7%) |  |
|  | Gangwon | 13 | 27 (+107.7%) | 0.25 (-98.1%) | 5 (-61.5%) | 27 (+107.7%) | 8 (-38.5%) | | 13 (0.0%) | 8 (-38.5%) | | 13 (0.0%) | 8 (-38.5%) | | 29 (+123.1%) |  |
|  | Gyeongbuk | 84 | 114 (+35.7%) | 22.75 (-72.9%) | 49 (-41.7%) | 116 (+38.1%) | 75 (-10.7%) | | 86 (+2.4%) | 66 (-21.4%) | | 92 (+9.5%) | 72 (-14.3%) | | 123 (+46.4%) |  |

^1)^ Values indicate the number of high – risk patches within the region under no change was applied to the parameter values.

^2)^ Values indicate the number of high – risk patches within the region when wild boars were allowed to move across two patches within a week.

^3)^ Values indicate the number of high – risk patches within the region with patch size set to 5km $\times$ 5km.

^4)^ Values indicate the reduction rate of high – risk patches to the baseline.


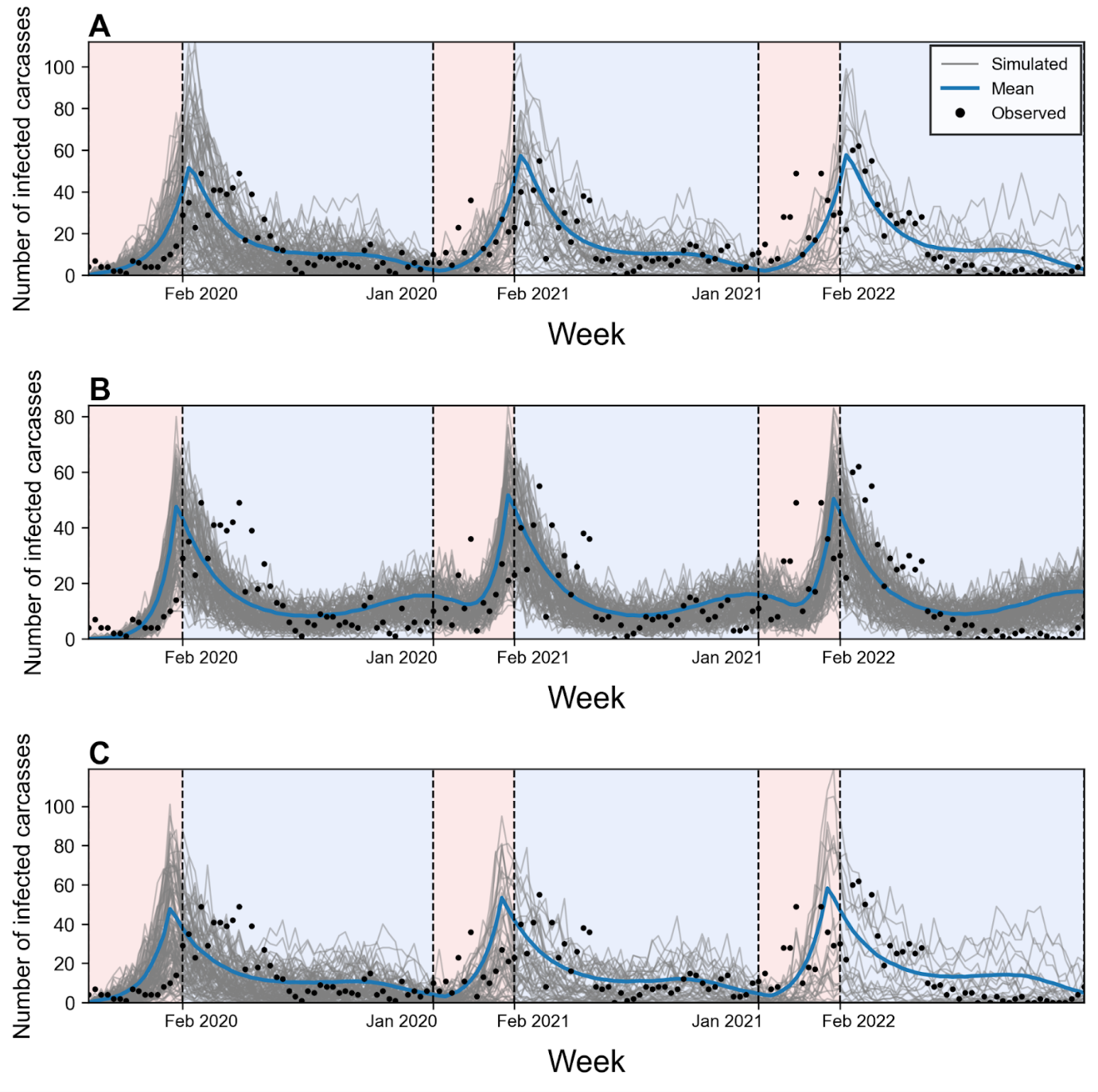
 **Supplementary Figure S1. Weekly ASF-infected carcass counts from 2019 to 2024, compared with simulated carcass trajectories generated using the seasonally varying carcass infectiousness-loss rate.** Stochastic simulated epidemic curve (gray-colored line), mean simulation trajectory averaged over 10,000 stochastics simulations (blue-colored line), and observed number of ASF-infected carcasses (dots). Vertical dashed lines indicate the transition points between increase and decrease phases. Red-shaded areas represent the increase phases and blue-shaded areas represent the decrease phases. Corresponding results using maximum and minimum values of $1/\delta_{c}(t)$ set to $\frac{37}{7}$ and $\frac{8}{7}$ (**A**), $\frac{30}{7}$ and $\frac{7}{7}$ (**B**), $\frac{365}{7}$ and $\frac{90}{7}$ (**C**).


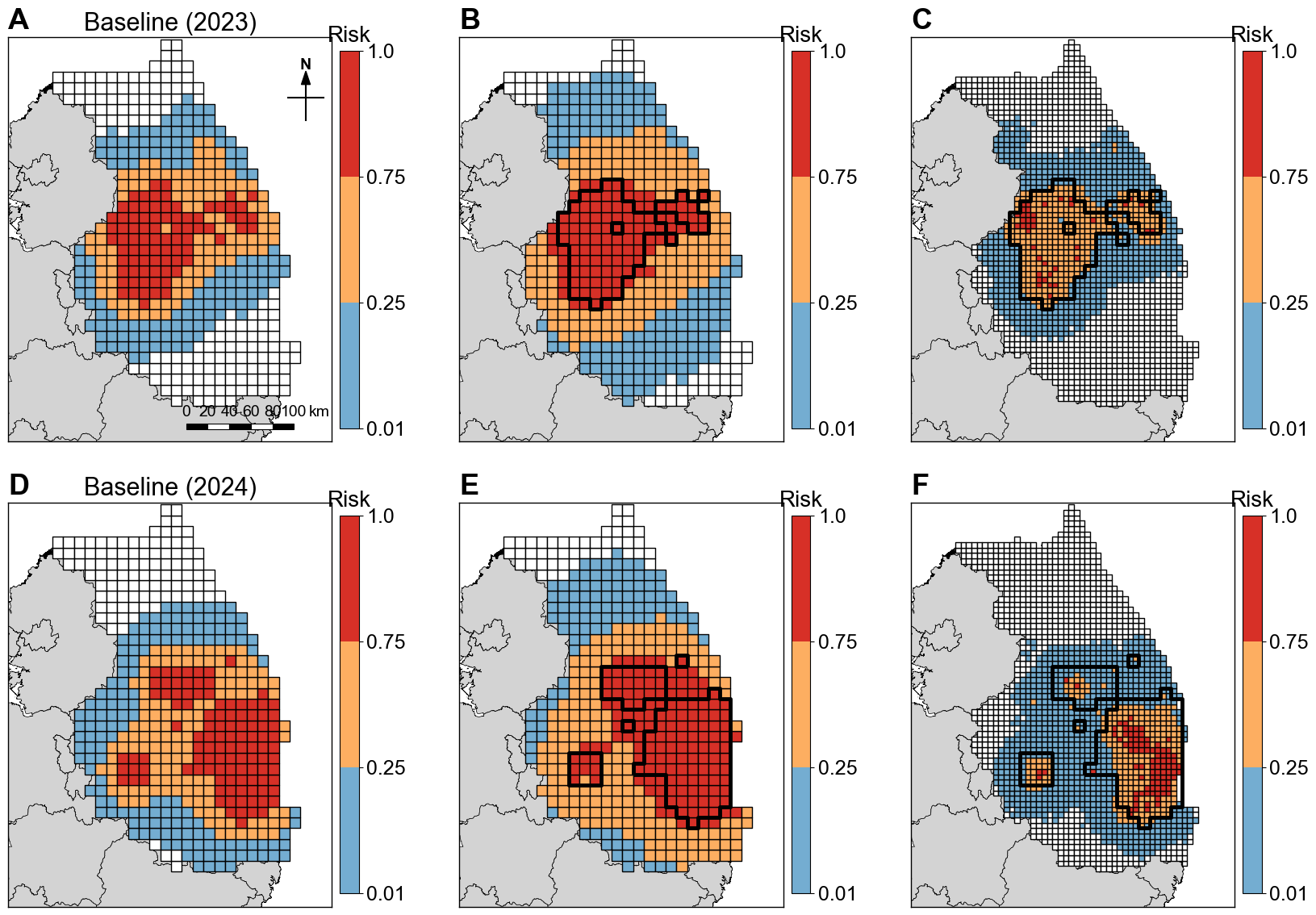


**Supplementary Figure S2. ASF outbreak risk using alternative movement-range and patch-size settings.** Simulated spatial distribution of outbreak risk using 10km $\times$ 10km patches with movement allowed to second-order neighboring cells for the 2023 (**A**) and 2024 (**B**) outbreak periods. Corresponding results using 5km $\times$ 5km patches with movement allowed to second-order neighboring cells for the 2023 (**C**) and 2024 (**D**) outbreak periods, respectively. Across these spatial configurations, hotspot locations remained consistent, whereas expanding the movement range increased the overall extent of predicted high-risk areas and adopting finer 5km $\times$ 5km patches produced a more detailed but spatially constrained risk footprint.


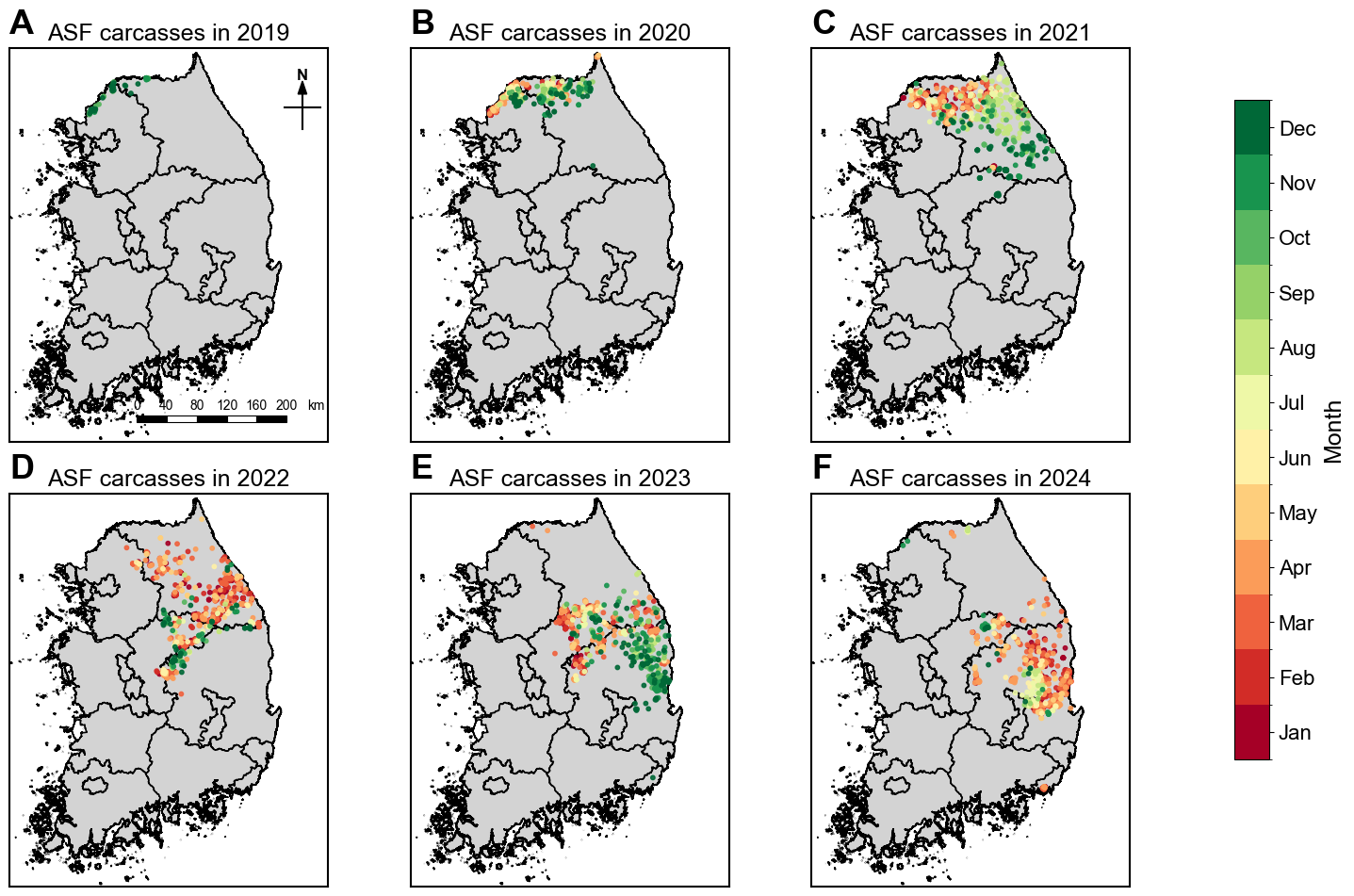


**Supplementary Figure S3. Spatiotemporal distribution of ASF-infected carcasses from 2019 to 2024. A–E.** Yearly distribution of ASF-infected carcasses from 2019 to 2024. Color represents the month of detection of ASF, shifting from red (early months) to green (later months).


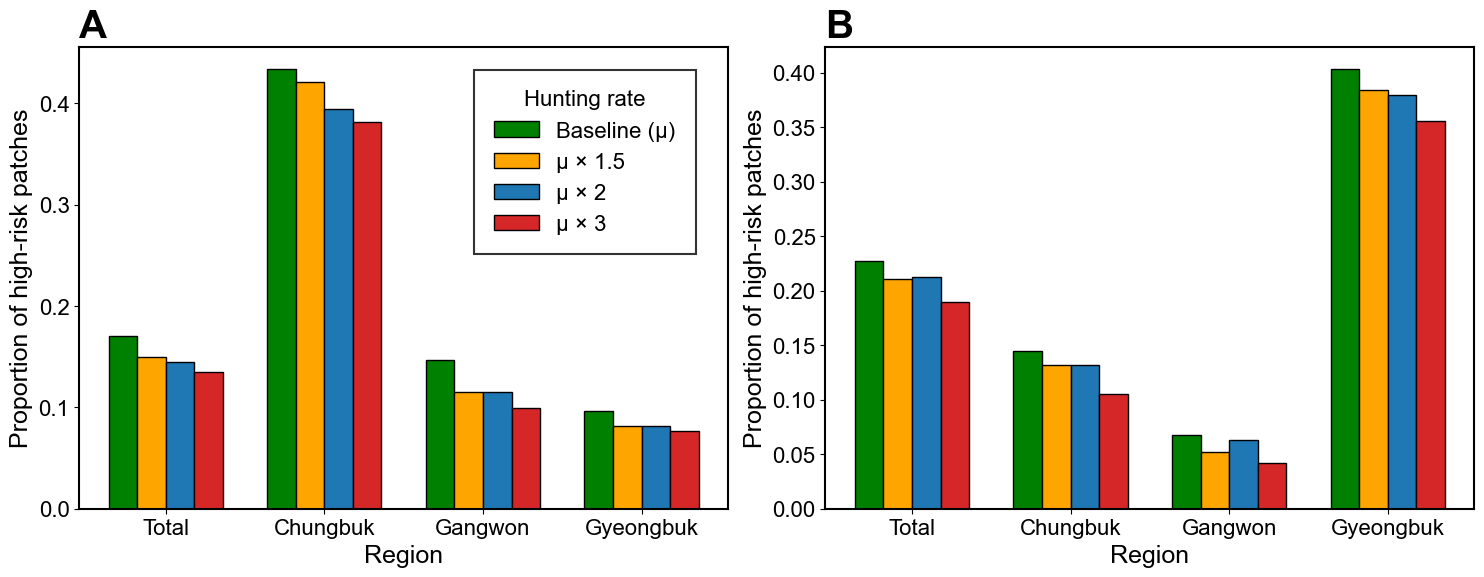


**Supplementary Figure S4. Proportion of infected patches by region according to hunting rate. A. and B.** Regional proportions of infected patches under different hunting rates for 2023 (**A**) and for 2024 (**B**).


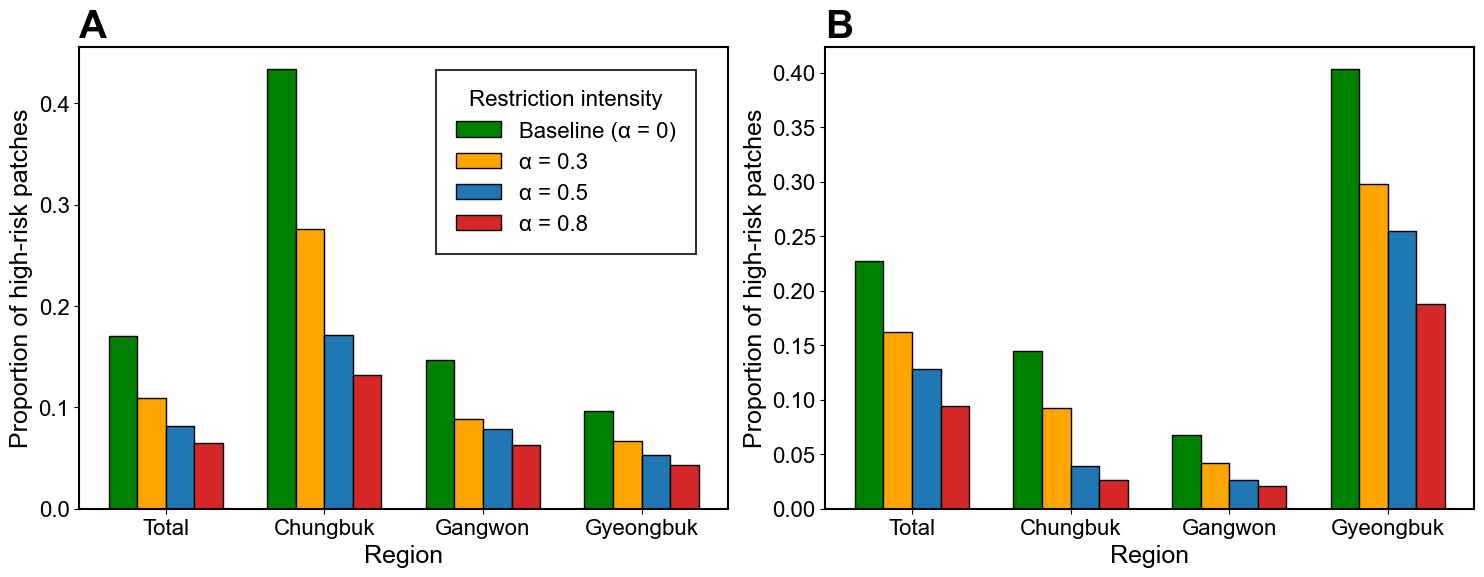


**Supplementary Figure S5. Proportion of infected patches by region according to restriction intensity (**$\alpha$**). A and B.** Regional proportions of infected patches under different restriction intensity for 2023 (**A**) and for 2024 (**B)**.

**References**

[1] M. Fischer, J. Hühr, S. Blome, F. J. Conraths, and C. Probst, “Stability of African Swine Fever Virus in Carcasses of Domestic Pigs and Wild Boar Experimentally Infected with the ASFV ‘Estonia 2014’ Isolate,” *Viruses*, vol. 12, no. 10, p. 1118, 2020.

[2] C. Probst, J. Gethmann, S. Amler, A. Globig, B. Knoll, and F. J. Conraths, “The Potential Role of Scavengers in Spreading African Swine Fever among Wild Boar,” *Scientific Reports*, vol. 9, no. 1, p. 11450, 2019.

[3] Ministry of Environment (2024), “*Improvement Measures for African Swine Fever (ASF) in Wild Boars*.” Available at: <https://www.me.go.kr/home/web/board/read.do?menuId=10525&boardMasterId=1&boardCategoryId=39&boardId=1672720> [Accessed July 24, 2025].

[4] C. Toïgo, S. Servanty, J.-M. Gaillard, S. Brandt, and E. Baubet, “Disentangling Natural from Hunting Mortality in an Intensively Hunted Wild Boar Population,” *Journal of Wildlife Management*, vol. 72, no. 7, pp. 1532–1539, 2008.

[5] P. van den Driessche, J. Watmough, “Reproduction Numbers and Sub-threshold Endemic Equilibria for Compartmental Models of Disease Transmission.” *Mathematical Biosciences*, vol. 180, no. 1–2, pp. 29–48, 2002.

[6] Ministry of Environment (2024), “*Wild Boar Capture in Gyeongbuk during Aug–Sep Increased by 89% Compared to Previous Year.*” Available at:<https://www.me.go.kr/home/web/board/read.do?menuId=10525&boardId=1703390&boardMasterId=1> [Accessed July 24, 2025].

[7] E. A. Dankwa, S. Lambert, S. Hayes, R. N. Thompson, and C. A. Donnelly, “Stochastic Modelling of African Swine Fever in Wild Boar and Domestic Pigs: Epidemic Forecasting and Comparison of Disease Management Strategies.” *Epidemics*, vol. 40, p. 100622, 2022.

[8] R. Engbert, M. M. Rabe, R. Kliegl, and S. Reich, “Sequential data assimilation of the stochastic SEIR epidemic model for regional COVID-19 dynamics.” *Bulletin of Mathematical Biology*, vol. 83, no. 1, p. 1, 2021.

[9] National Institute of Ecology (2025), “*Survey Data from the 5th National Natural Environment Survey, National Institute of Ecology.*” Available at: <https://www.data.go.kr/data/15138804/fileData.do?recommendDataYn=Y> [Accessed July 24, 2025].

[10] Ministry of Environment (2024), “*Improvement Measures for African Swine Fever (ASF) in Wild Boars*.” Available at: <https://www.me.go.kr/home/web/board/read.do?menuId=10525&boardMasterId=1&boardCategoryId=39&boardId=1672720> [Accessed July 24, 2025].

[11] Ministry of Ecology (2023), “*The nationwide wild boar population density is 1.1 individuals per square kilometer.*” Available at: https://www.me.go.kr/home/web/board/read.do?menuId=10525&boardId=1596710&boardMasterId=1 [Accessed July 24, 2025].

[12] National Geographic Information Institute (2025), “*National Land Map*”. <https://map.ngii.go.kr/ms/map/NlipMap.do?tabGb=total> [Accessed July 24, 2025].

[13] Korea Meteorological Administration (2025), “*Open Meteorological Data Portal*.” Available at: <https://data.kma.go.kr/cmmn/main.do;jsessionid=ktFt3vps8Tbd1zYhO5lzLEDeNhhjfryMt04aYvhy5STulvkpvKj3sUgHstBrrrH0.was01_servlet_engine5> [Accessed July 24, 2025].

[14] D. S. Choi, Y. J. Lee, M. J. Ko., “Utilization and verification of inverse distance weighting (IDW) interpolation technology for predicting solar radiation of photovoltaic system.” *KIEAE Journal*, 22(1), 5-12.

[15] S. N. Kim, W. K. Lee, K. I. Shin, M. Kafatos, D. J. Seo, and H. B. Kwak (2010). “Comparison of spatial interpolation techniques for predicting climate factors in Korea”. *Forest Science and Technology*, vol. 6, no.2, pp. 97-109, 2010.

[16] Didan, K. (2021), “MODIS/Terra Vegetation Indices 16-Day L3 Global 250 m SIN Grid V061.” NASA Land Processes Distributed Active Archive Center. Available at: https://doi.org/10.5067/MODIS/MOD13Q1.061 [Accessed October 14, 2025].

[17] WorldPop (2018), “*Global High Resolution Population Denominators Project.*” Available at: https://dx.doi.org/10.5258/SOTON/WP00647 [Accessed July 24, 2025].

[18] J. Rew, Y. Cho, J. Moon, and E. Hwang, "Habitat suitability estimation using a two-stage ensemble approach." *Remote Sensing*, 2020.

[19] S. Liu, Y. Tian, Y. Liu, I. D. Alabia, J. Cheng, and S. I. Ito, “Development of a prey-predator species distribution model for a large piscivorous fish: A case study for Japanese Spanish mackerel Scomberomorus niphonius and Japanese anchovy Engraulis japonicus.” *Deep Sea Research Part II: Topical Studies in Oceanography*, 2023.

[20] F. Ghareghan, G. Ghanbarian, H. R. Pourghasemi, “Prediction of habitat suitability of Morina persica L. species using artificial intelligence techniques.” *Ecological Indicators*, 2020.

[21] A. M. Gormley, D. M. Forsyth, P. Griffioen, M. Lindeman, D. S. L. Ramsey, M. P. Scroggie, and L. Woodford, “Using Presence‐Only and Presence–Absence Data to Estimate the Current and Potential Distributions of Established Invasive Species.” *Journal of Applied Ecology*, vol. 48, no. 1, pp. 25–34.

[22] J. Elith, C. H. Graham, R. P. Anderson, M. Dudík, S. Ferrier, A. Guisan, R. J. Hijmans, F. Huettmann, J. R. Leathwick, A. Lehmann, J. Li, L. G. Lohmann, B. A. Loiselle, G. Manion, C. Moritz, M. Nakamura, Y. Nakazawa, J. McC. M. Overton, A. T. Peterson, S. J. Phillips, K. Richardson, R. Scachetti-Pereira, R. E. Schapire, J. Soberón, S. Williams, M. S. Wisz, and N. E. Zimmermann, “Novel Methods Improve Prediction of Species’ Distributions from Occurrence Data.” *Ecography*, vol. 29, no. 2, pp. 129–151, 2006.

[23] A. Jiménez-Valverde and J. M. Lobo, “Threshold criteria for conversion of probability of species presence to either–or presence–absence.” *Acta Oecologica*, vol. 31, no. 3, pp. 361–369, 2007.

[24] O. Allouche, A. Tsoar, and R. Kadmon, “Assessing the accuracy of species distribution models: prevalence, kappa and the true skill statistic (TSS).” *Journal of Applied Ecology*, vol. 43, no. 6, pp. 1223–1232, 2006.

[25] C. Liu, G. Newell, and M. White, “On the selection of thresholds for predicting species occurrence with presence-only data.” *Ecology and Evolution*, vol. 6, no. 1, pp. 337–348, 2016.

[26] M. Dong, J. Yang, Y. Fu, T. Fu, Q. Zhao, X. Zhang, Q. Xu, and W. Zhang, "Distribution of Suitable Habitats for Soft Corals (Alcyonacea) Based on Machine Learning." *Journal of Marine Science and Engineering*, 2024.

[27] T. Yang, L. Xingyu, H. Zhiqiang, "Predicting the effects of climate change on the suitable habitat of Japanese Spanish mackerel (Scomberomorus niphonius) based on the species distribution model." *Frontiers in Marine Science*, 2020.

[28] M. O. Park, “Spatial Distribution and Habitat Characteristics of ‘Wild Boar (Sus scrofa)’.” *Journal of the Korea Institute of Garden Design*, *8*(3), vol. 8, no.3, pp. 196-207, 2022.

[29] C. Toïgo, S. Servanty, J.-M. Gaillard, S. Brandt, and E. Baubet , “Disentangling Natural from Hunting Mortality in an Intensively Hunted Wild Boar Population,” *Journal of Wildlife Management*, vol. 72, no. 7, pp. 1532–1539, 2008.

[30] J. H. Han, D. S. Yoo, S. I. Pak, E. T. Kim, "Understanding the Transmission of African Swine Fever in Wild Boars of South Korea: a Simulation Study for Parameter Estimation." *Transboundary & Emerging Diseases*, vol. 69, no. 4, e1101–e1112, 2022.

[31] R. A. Taylor, T. Podgórski, R. R. L. Simons, S. Ip, P. Gale, L. A. Kelly, and E. L. Snary, "Predicting Spread and Effective Control Measures for African Swine Fever-Should We Blame the Boars?" *Transboundary & Emerging Diseases*, vol. 68, no. 2, pp. 397–416, 2021.

[32] O. Keuling, K. Lauterbach, N. Stier, M. Roth, “Hunter Feedback of Individually Marked Wild Boar Sus scrofa L.: Dispersal and Efficiency of Hunting in Northeastern Germany.” *European Journal of Wildlife Research*, vol. 56, no. 2, pp. 159–167, 2010.

[33] European Space Agency (2022), “*ESA WorldCover 10 m 2021 v200.*” Available at: https://doi.org/10.5281/zenodo.7254221 [Accessed July 24, 2025].

[34] Abbott, S., Hellewell, J., Thompson, R. N., Sherratt, K., Gibbs, H. P., Bosse, N. I., ... & Funk, S. Estimating the time-varying reproduction number of SARS-CoV-2 using national and subnational case counts. Wellcome Open Research, 5(112), 112, 2020
